# Cobalt Ferrite Nanoparticles for Tumor Therapy: Effective Heating versus Possible Toxicity

**DOI:** 10.3390/nano12010038

**Published:** 2021-12-23

**Authors:** Anastasiia S. Garanina, Alexey A. Nikitin, Tatiana O. Abakumova, Alevtina S. Semkina, Alexandra O. Prelovskaya, Victor A. Naumenko, Alexander S. Erofeev, Peter V. Gorelkin, Alexander G. Majouga, Maxim A. Abakumov, Ulf Wiedwald

**Affiliations:** 1 National University of Science and Technology «MISiS», 119049 Moscow, Russia; anastasiacit@gmail.com (A.S.G.); nikitin.aa93@mail.ru (A.A.N.); sasha.prelove@gmail.com (A.O.P.); erofeev@polly.phys.msu.ru (A.S.E.); alexander.majouga@gmail.com (A.G.M.); abakumov1988@gmail.com (M.A.A.); 2 Skolkovo Institute of Science and Technology, 121205 Moscow, Russia; sandalovato@gmail.com; 3 Department of Medical Nanobiotechnology, Russian National Research Medical University, 117997 Moscow, Russia; alevtina.semkina@gmail.com; 4 V. Serbsky National Medical Research Center for Psychiatry and Narcology, 119034 Moscow, Russia; naumenko.vict@gmail.com; 5 Department of Chemistry, Lomonosov Moscow State University, 119991 Moscow, Russia; 6 Medical Nanotechnology LLC, Skolkovo Innovation Center, 121205 Moscow, Russia; peter.gorelkin@gmail.com; 7 D. Mendeleev University of Chemical Technology of Russia, 125047 Moscow, Russia; 8 Center for Nanointegration Duisburg-Essen, Faculty of Physics, University of Duisburg-Essen, 47057 Duisburg, Germany

**Keywords:** cobalt ferrite nanoparticles, biocompatibility, magnetic properties, controlled magnetic hyperthermia, tumor therapy

## Abstract

Magnetic nanoparticles (MNPs) are widely considered for cancer treatment, in particular for magnetic hyperthermia (MHT). Thereby, MNPs are still being optimized for lowest possible toxicity on organisms while the magnetic properties are matched for best heating capabilities. In this study, the biocompatibility of 12 nm cobalt ferrite MNPs, functionalized with citrate ions, in different dosages on mice and rats of both sexes was investigated for 30 days after intraperitoneal injection. The animals’ weight, behavior, and blood cells changes, as well as blood biochemical parameters are correlated to histological examination of organs revealing that cobalt ferrite MNPs do not have toxic effects at concentrations close to those used previously for efficient MHT. Moreover, these MNPs demonstrated high specific loss power (SLP) of about 400 W g^−1^. Importantly the MNPs retained their magnetic properties inside tumor tissue after intratumoral administration for several MHT cycles within three days. Thus, cobalt ferrite MNPs represent a perspective platform for tumor therapy by MHT due to their ability to provide effective heating without exerting a toxic effect on the organism. This opens up new avenues for smaller MNPs sizes while their heating efficiency is maintained.

## 1. Introduction

Cancer ranks second in mortality worldwide and is also associated with a severely declining life quality. Currently, new promising approaches to the malignant neoplasms therapy are being developed, among which considerable attention is paid to controlled magnetic hyperthermia (MHT) [1]. It is provided by magnetic nanoparticles (MNPs), which generate heat under a radio-frequency alternating magnetic field (AMF) exposure. Thus, the accumulation of MNPs in the tumor and AMF treatment result in local and controlled tumor heating to temperatures above 42 °C eventually leading to cell death. 

The effectiveness of MHT depends on the chemical composition, size, shape, anisotropic magnetic properties, and stability of MNPs that are used for this purpose [2,3,4]. Iron oxide-based MNPs are actively studied as a perspective material for MHT application [2,5]. They exhibit high magnetization, but low magnetic anisotropy, good control over size and shape; however, they are prone to aggregation and oxidation. Therefore, iron oxide MNPs should be covered by protective shells, which can result in decreasing heating efficiencies [6]. Thereby, in recent years, ferrite-based MNPs have been actively explored for biomedical application, in particular for MHT [7,8,9,10]. Ferrite MNPs allow to tailor magnetization and magnetic anisotropy in a wide range by slight stoichiometry variations. This ultimately leads to better matching on the MHT parameters and also smaller MNPs can be employed for MHT as compared with the commonly used 20–25 nm iron oxide MNPs [7]. To increase the colloidal stability, they are usually coated with citrate ions, but such modification does not affect the heating ability of MNPs [11]. Thus, in terms of physical and chemical properties, such spinel ferrite MNPs are promising candidates for their use in MHT cancer therapy.

In addition to their magnetic properties, the biocompatibility of MNPs plays a crucial and decisive role for applications in biomedicine. Early reports found significant toxicity of MNPs in cell environments [12,13,14]. However, these studies were mainly performed in in vitro conditions, because toxicological investigations using animal models are expensive and time consuming. With the development of nanotechnology, the biocompatibility of MNPs has received increased attention. Currently, detailed analysis of some types of iron oxide MNPs are carried out. It was shown that one of the effective ways to reduce MNPs toxicity to the body is to cover them with different coatings [15]. Thus, nowadays the application of iron oxide MNPs for cancer diagnosis, cancer hyperthermia, and iron deficiency anemia is approved by US Food and Drug Administration (FDA) [16]. 

The biocompatibility investigation of ferrite-based MNPs is, however, only addressed in a few studies, which focus on different cell lines [17]. The most studied ferrite-based MNPs are cobalt ferrites [18]. Nevertheless, even for them, the number of detailed biocompatibility studies described in the literature is highly limited. Researchers mainly addressed the toxicity of cobalt-ferrite MNPs after intravenous administration [19]. The MNPs concentrations used in the latter study are suitable for diagnostic investigations of malignant neoplasms using MRI, however, are not able to provide the required heating of the tumor tissue during MHT. 

In the present study we focus on the in vivo investigation of cobalt ferrite MNPs and their possible toxicity at higher concentrations suitable for MHT. Animals’ weight and behavior change, leukocyte formula, blood biochemical parameters, and organ histology were analyzed after intraperitoneal (i.p.) cobalt ferrite MNPs injection at three different concentrations for up to 30 days after administration to mice and rats of both sexes. We show that MNPs at concentrations providing the required heat in MHT, and even higher doses do not have a significant toxic effect on the animal organism. The profit of cobalt ferrites is further underlined by their high specific loss power (SLP) at reduced size as compared to magnetite and retaining magnetic properties inside tumor tissue for at least three days that was necessary for the full course of tumor therapy by MHT. A first scenario for MNPs degradation and dissolution in living organisms is drawn. 

## 2. Materials and Methods

### 2.1. Synthesis of Cobalt Ferrite Nanoparticles 

Synthesis of cobalt ferrite (CoFe_2_O_4_) MNPs has been described in our previous study [20]. Briefly, 4 mL of 2.5 M FeCl_3_ (≥98%, Sigma-Aldrich, Saint Louis, MO, USA) was mixed with 4 mL of 1.25 M CoCl_2_·6H_2_O (98%, Plasmotherm, Moscow, Russia) and this solution was added dropwise to 200 mL of 1 M NaOH (≥98%, Sigma-Aldrich, Saint Louis, MO, USA), and preheated to the boiling point. The mixture was kept at reflux for 30 min, followed by cooling to room temperature. MNPs were separated by centrifugation (10 min, 6000 rpm) and washed with deionized water (3 × 200 mL). Then, 500 mL of 2 M HNO_3_ (65%, Sigma-Aldrich, Saint Louis, MO, USA) and 30 mL of 0.35 M Fe(NO_3_)_3_ (≥98%, Reactivtorg) were added to MNPs. The obtained mixture was heated to boiling and maintained for 1 h at a vigorous stirring. MNPs were separated by centrifugation (10 min, 6000 rpm) after cooling the mixture to room temperature and washed with 2 M solution of HNO_3_ (3 × 200 mL). The precipitate of MNPs was redispersed in 200 mL of deionized water, followed by the addition of 40 mL of 0.2 M citric acid (98%, Sigma-Aldrich, Saint Louis, MO, USA). After incubation for 10 min the pH of the mixture was adjusted to 7.4 using 1 M NaOH. Finally, the colloidal solution of MNPs was purified from excess of citrate-ions using centrifuge filters with 100 kDa pore size (30 min, 6000 rpm). Sediment of MNPs were redispersed in deionized water and passed through the 0.2 μm syringe filter to remove any aggregates and impurities.

For the Mössbauer study of the MNPs stability to biotransformation, cobalt ferrite MNPs enriched in ^57^Fe isotope were synthesized. The synthesis followed the above protocol using ^57^FeCl_3_ (≥95% ^57^Fe content) as a precursor.

### 2.2. Transmission Electron Microscopy (TEM)

The size of MNPs was analyzed by JEM-1400 (120 kV) transmission electron microscope (JEOL, Tokyo, Japan). For this, 3 µL of MNPs solution in distilled water (0.1 mg mL^−1^) was placed on a carbon-coated cooper grid and left to dry. The size of MNPs was measured from the obtained images by ImageJ software. At least 1000 MNPs were analyzed. 

### 2.3. Scanning Electron Microscopy (SEM) and Energy-Dispersive X-ray Spectroscopy (EDX)

The morphology and elemental analysis of the obtained MNPs were studied using a JEOL JSM-IT 500 SEM (Tokyo, Japan) at 20 kV acceleration voltage. The elemental analysis was performed using energy-dispersive X-ray spectroscopy. Powder of MNPs was placed onto the surface of double-sided carbon tape.

### 2.4. Mössbauer Spectroscopy

^57^Fe Mössbauer absorption spectra were measured with electro-dynamical type spectrometer CMS-1104Em (Rostov-on-Don, Russia), working in the constant acceleration mode. The spectra were measured both at liquid nitrogen temperature and at ambient temperature. ^57^Co in a rhodium matrix was used as a source of the resonant gamma-irradiation. Isomer shifts were determined in relation to the absorption line of α-Fe. The analysis of all Mössbauer spectra was carried out in the manystate relaxation model formalism with assumption of two sublattices, which corresponds to the two sextet components in the spectra [21].

### 2.5. Vibrating Sample Magnetometry

Magnetometry at variable temperature (2–300 K) and fields (±9 T) was measured in a Quantum Design PPMS DynaCool system (Quantum Design, San Diego, CA, USA). For this, about 10 mg dried powder of the CoFe_2_O_4_ MNPs was put in synthetic capsules.

### 2.6. Cell Lines

CT26 (mouse colon carcinoma cells) and RAW 264.7 (murine macrophage cells) were purchased from the American Type Culture Collection (ATCC, Manassas, VA, USA). Cells were cultured in RPMI-1640 or DMEM medium (ThermoFisher Scientific, Waltham, MA, USA), correspondingly, supplemented with 10% fetal bovine serum (FBS) and 2 mM L-glutamine (ThermoFisher Scientific, Waltham, MA, USA). The culture medium for CT26 cells was also supplemented with 10 mM HEPES (Helicon, Moscow, Russia), and 1 mM sodium pyruvate (ThermoFisher Scientific, Waltham, MA, USA). Cells were maintained at 37 °C in a humidified incubator (Sanyo, Osaka, Japan) supplied with 5% CO_2_.

### 2.7. Nanoparticle Stability Assay after Incubation with Cells

RAW 264.7 cells were seeded in T75 culture flasks (EasyFlask) in concentration of 60,000 cells per mL and cultured for 48 h. Then, the culture medium was replaced with a new one containing 30 μg mL^−1^ of Co^57^Fe_2_O_4_ MNPs, and the cells were further incubated for 72 h at 5% CO_2_ and 37 °C. Later, one half of cells was detached, thoroughly washed with a buffer solution by centrifugation at least five times, and dried using a vacuum rotary evaporator. The second half of the cells containing MNPs was cultivated for another 72 h and then prepared for examination in the same way. As a result, two samples of dried cells containing MNPs for Mössbauer spectroscopy study were obtained: with an incubation period of 3 and 6 days. Additionally, a powder sample of the initial Co^57^Fe_2_O_4_ MNPs was dried and prepared.

### 2.8. Toxicity Studies of Nanoparticles

In vivo toxicity studies of MNPs were carried out on six-week-old female (n = 25) and male (n = 25) BALB/c mice with weight 18–20 g, as well as on five-week-old female (n = 25) and male (n = 25) Wistar rats with weight 180–200 g obtained from Andreevka Animal Center (Andreevka, Russia). All animal experiments were approved by N.I. Pirogov Russian National Research Medical University bioethical committee (protocol ### 25/2017, 26/2017, 01/2020). Tumor-free animals were investigated to avoid the possible impact of malignant neoplasm on their state and behavior. The solution of MNPs in DPBS (pH 7.4; ThermoFisher Scientific, Waltham, MA, USA) was i.p. injected to experimental animals at different concentrations in the morning before feeding. Animals injected with DPBS were taken as vehicle control and intact mice and rats—as control. Three different concentrations of MNPs were studied for mice: doses of 230 (minimal), 1300 (intermediate), and 3000 mg kg^−1^ (maximum). The calculation of similar doses for rats was carried out according to the formula of interspecies dose transfer, based on the size of experimental animals body surface area:

X_r_ = (X_m_ × K_m_)/K_r_, (1)

where X_r_—dose for rats, mg kg^−1^; X_m_—dose for mice, mg kg^−1^; K_m_—mice coefficient; K_r_—rats’ coefficient. In our case, K_m_ is equal to 3 (for mice with an average weight of 20 g) and K_r_ = 6.5 (for rats with an average weight of 200 g) [22]. As a result, the following doses for rats were studied: 106.2 mg kg^−1^, 600, and 1384.6 mg kg^−1^. 

The change in body weight and animals’ behavior was studied for 30 days. On the first day after MNPs injection, the animals were under continuous ten-hour observation. After 30 days, animals were anesthetized by i.p., injection of 50 mg kg^−1^ Zoletil and 5 mg kg^−1^ xylazine. When animals reached the stage of deep anesthesia, the blood and organs were taken for analysis. Blood was collected through cardiac puncture into syringes containing 2% EDTA. Each animal underwent a clinical (automatic analyzer Mindray Labio, Shenzhen, China) and biochemical (Exell22 hematology analyzer, Drew Scientific, Inc., Florida, USA) blood test. Organs and tissues were harvested from all animals and fixed in a separate vial in 10% buffered formalin for 24 h. Then they were washed for one day with running water and dehydrated according to the following scheme: incubation in 70° alcohol for 1 h, 96° alcohol—1.5 h, 100° alcohol—3 h. Later, samples were incubated for 40 min in chloroform and placed in paraplast overnight at 56 °C for impregnation. After that, the samples were placed in a fresh portion of paraplast and left at room temperature for polymerization. Histological preparations of organ sections (4 μm thick) were performed on an STP 120-1 machine for histological tissue processing (MIKROM). Obtained samples of blood smears and organ sections were stained with hematoxylin and eosin and analyzed by Leica light microscope (Wetzlar, Germany). 

### 2.9. Animals and Tumor Model

CT26 tumor was established by subcutaneous (s.c.) injection of 1.6 × 10^6^ cells into the right hind flank of a BALB/c female mice.

### 2.10. Controlled MNPs Hyperthermia

When tumors reached ~30 mm^2^ (8 days after cell implantation), mice were anesthetized, and MNPs solution in DPBS (100 μL with concentration 60 mg mL^−1^ by iron) was intratumorally (i.t.) injected. For controlled magnetic hyperthermia, anesthetized mouse was placed in the AMF generator TOR UltraHT (NanoMaterials, Tambov, Russia) designed for animals’ exposure to AMF. Following parameters of AMF were applied—f = 261 kHz, B = 15–25 mT—to reach and keep a constant temperature of 46–48 °C. The temperature in the tumor and contralateral skin area was measured by a Seek Thermal camera. AMF was applied for 30 min.

### 2.11. MNPs Magnetic Properties Study after Intratumoral Injection and Magnetic Hyperthermia

After i.t. injection and MHT tumor-bearing mice were anesthetized and sacrificed. Tumors were surgically removed, fixed with 10% neutral formalin, and impregnated with paraplast as described above. An about 2 × 2 × 2 mm^3^ piece of tumor tissue with MNPs (identified by the black color) was cut off and examined by a vibration sample magnetometry.

### 2.12. Statistical analysis

Statistical processing of the obtained data was carried out using nonparametric criteria recommended for small sample sizes. To evaluate the results, the mean, median, standard error of the mean (±SE), standard deviation (±SD), minimum (Min) and maximum (Max) values, interquartile percentiles P_25%_ and P_75%_ were given. To determine the significance of differences between animals groups, the Kruskal–Wallis one-way ANOVA test was used. Statistical analysis was performed using Statistica 13.0 software (TIBCO Software Inc., Palo Alto, CA, USA). The analysis was performed for each species and sex of animals separately. Comparison of the indicators was carried out relative to the indicators of the control animals’ group. Data of animals’ weight change were analyzed using the unpaired *t*-test in GraphPad Prism 5 software. *p* values < 0.05 were considered significant.

## 3. Results

### 3.1. Cobalt Ferrite MNPs Characteristic

**Figure 1:**
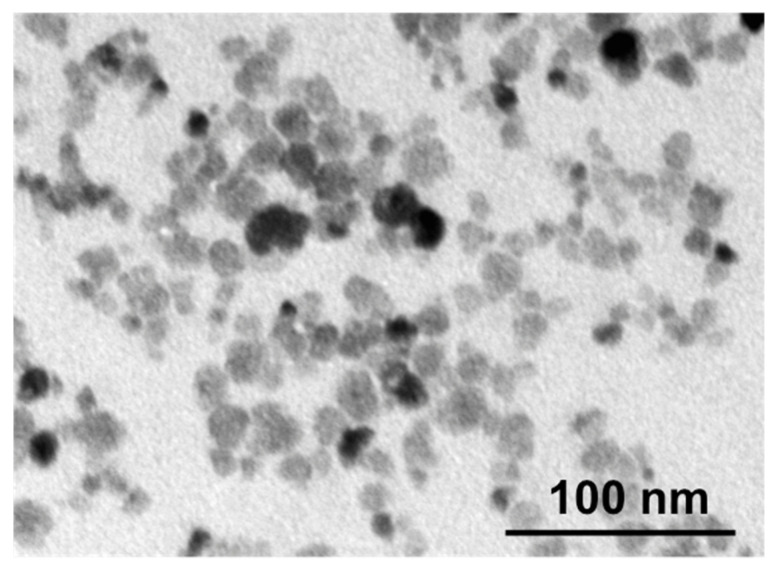
TEM micrograph of investigated cobalt ferrite MNPs.

The citrate-functionalized CoFe_2_O_4_ MNPs used in this study have been described in detail in our previous work [20]. Briefly, the mean value of the core diameter revealed by TEM was 12 ± 4 nm (Figure 1). EDX analysis revealed Co, Fe, and O contents of 11.4 ± 1.1 at-%, 25.5 ± 1.3 at-%, and 63.0 ± 1.5 at-%, respectively. Higher O content is often found due to storage in air, and more importantly, structural investigations by X-ray diffraction have proven the inverse spinel structure of CoFe_2_O_4_ MNPs [23]. 

Intracellular biotransformation of iron-based MNPs is a characteristic process for living organisms, because iron is an important element for them [24]. Since the MNPs are planned to be injected in tumors (for MHT therapy), where they can be gradually captured by specialized phagocytes, mainly macrophages, their chemical and phase stability was studied in biological environment (namely during incubation with RAW 264.7 cells). Figure S1 presents ^57^Fe-Mössbauer absorption spectra of Co^57^Fe_2_O_4_ MNPs. At liquid nitrogen temperature, the spectrum of initial MNPs is a well-resolved sextet with an asymmetric line shape. At room temperature, the particle spectrum is a broadened relaxation sextet with a nearly symmetric line shape. This behavior is typical for an ensemble of superparamagnetic cobalt ferrite MNPs [25]. It is well-known that the changes caused by biotransformation of iron oxide MNPs are reflected as a transformation of the hyperfine structure. A sign of MNPs biotransformation is the simultaneous appearance of a doublet component in the spectrum, which corresponds to the formation of paramagnetic forms of iron, and a change in the parameters of the sextet components of the spectrum, which corresponds to the degradation of the MNPs crystal lattice [26]. However, the hyperfine structure of synthesized Co^57^Fe_2_O_4_ MNPs spectra remains virtually unchanged even after 6 days of incubation with macrophage cells. This result indicates the absence of measurable intracellular biotransformation of investigated MNPs.

The study of magnetostatic properties of synthesized MNPs powder yielded a saturation magnetization of M_S_ = 65.3 Am^2^ kg^−1^ at B = 9 T and T = 5 K and 48.1 Am^2^ kg^−1^ (46.5 Am^2^ kg^−1^) at B = 3 T and T = 300 K (318 K). The mean superparamagnetic blocking temperature is T_B_ = 72 K as deduced from Sharrock fitting of the coercive field as function of temperature with a broad distribution showing irreversibilities of the ZFC/FC curves up to 250 K [23]. For frequencies in the few 100 kHz range the MNPs are magnetically blocked at physiological temperatures. The combination of these magnetic features make the CoFe_2_O_4_ MNPs very suitable for MHT. The MNPs show SLP value of about 400 W g^−1^ at 375 kHz and B = 25 mT and were successfully used for 30 min tumor heat treatment at elevated temperature in mice in slightly different conditions [20]. 

### 3.2. MNPs Toxicity Study

To generate the required heating for tumor therapy, the injected MNPs concentrations can be highly variable depending on nanoparticles physicochemical properties and route of administration. Despite the fact that MNPs intravenous (i.v.) administration is minimally invasive and can deliver MNPs to non-localized cancer tumors, such way of MNPs injection does not allow reaching heating above 40 °C. The calculation of the MNPs concentration required to generate desired intratumoral temperatures during hyperthermia after MNPs i.v. administration showed that it should reach 1700 mg of iron per kg of body weight [27]. Bubnovskaya et al. demonstrated effective heating of mice tumors after i.t. administration of MNPs at a dose of 200 mg kg^−1^. Wherein, the tolerable dose of MNPs upon i.p. injection was 300 mg kg^−1^ [28]. Thus, in order to use the cobalt ferrite MNPs for MHT, their potential toxicity to the body should be addressed first. In this regard we investigated three different MNPs concentrations for mice—230 mg kg^−1^ (minimal dose, which is close to therapeutic [20] and provide effective heating), 1300 mg kg^−1^ (intermediate), and 3000 mg kg^−1^ (maximum) as well as for rats—106, 600, and 1384.6 mg kg^−1^ (equivalent doses according to the interspecies transfer formula), respectively. Acute toxicity of MNPs was studied on 50 mice and 50 rats (males and females equally). MNPs were once injected intraperitoneally. I.p. injection was chosen because: (1) MNPs were designed for the therapy of tumors, including the neoplasms of internal organs, from where nanoparticles can migrate to surrounding tissues and organs, affecting the liver, kidneys, spleen, etc.; (2) since a tumor is a neoplasm with a well-developed system of blood vessels, the way of MNPs administration during a study in healthy animals should provide a model for the MNPs interaction with blood vessels, which is adequately reproduced only with i.p. injection.

It was revealed that maximum dose of MNPs resulted in 40% death of male mice by the eighth day after injection. However, none of the studied MNPs concentrations led to the death of female mice. In the case of rats, maximum dose caused 20% death of female animals, and intermediate dose resulted in 20% death of male animals (see Table S1).

#### 3.2.1. Animals Weight Change after MNPs Injection

**Figure 2:**
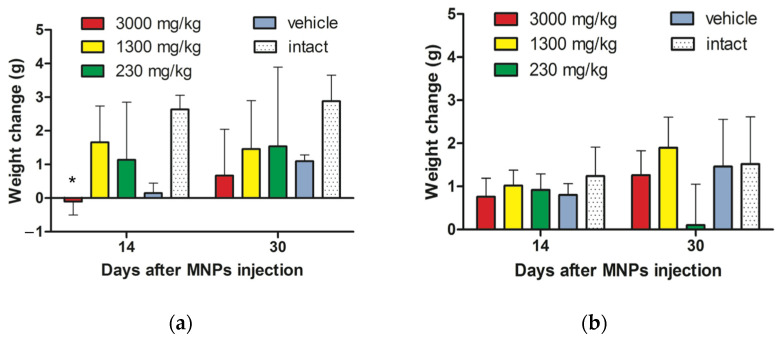

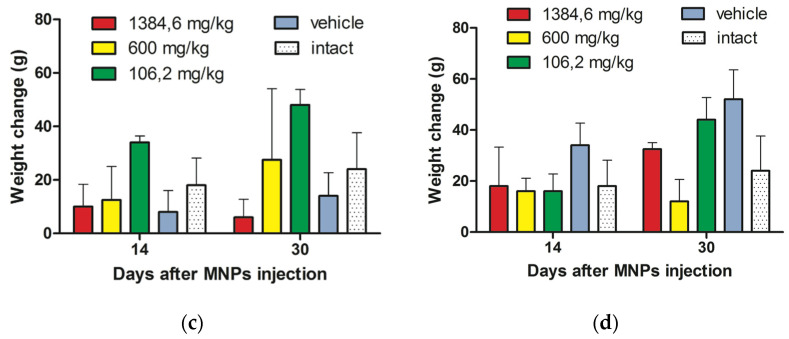
Animals weight change in the study of cobalt ferrite MNPs acute toxicity after i.p. injection. (**a**) Normalized body weight comparison between male mice from five experimental groups; (**b**) Normalized body weight comparison between female mice from different experimental groups; (**c**) Normalized body weight comparison between male rats from experimental groups; (**d**) Normalized body weight comparison between female rats from experimental groups. Results are shown as means ± SEM. * *p* < 0.05 (unpaired *t*-test).

Investigation of animals’ weight change after cobalt ferrite MNPs injection during 30 days showed the loss in body weight of male mice, administered with maximum dose of MNPs, compared to intact animals, which was especially pronounced on the 14th day after CoFe_2_O_4_ MNPs injection (* *p* < 0.05, unpaired *t*-test) (Figure 2a). In other groups of male and female mice, no statistically significant differences in body weight change were revealed compared to intact animals for the entire observation period (Figure 2a,b). Moreover, we analyzed the number (in % by groups) of male and female mice with a negative value of weight gain. As a result, statistically significant differences were revealed between the groups of heterosexual mice that were injected by a single dose of 3000 mg kg^−1^ (p_K-W_ < 0.05, Kruskal–Wallis one-way ANOVA test) and 1300 mg kg^−1^, in other groups no differences were found (Figure S2). Thus, it can be concluded that male mice are most susceptible to a toxic effect following injected MNPs that manifests in the form of body weight negative dynamics compared to female animals. This fact is especially pronounced when MNPs are injected at maximum concentration.

Statistical analysis of the male as well as female rats weight change did not reveal significant differences at all doses compared with the intact group (Figure 2c,d). Investigation of the number of male and female rats with a negative value of weight gain (in % by groups) also demonstrated no statistically significant differences in weight loss by gender (Figure S3). 

#### 3.2.2. Animals’ Behavior Changes after MNPs Injection

To assess the general condition of animals after MNPs injection, different indicators of their behavioral response and the general condition were compared with the norms for intact animals (see Table S2). Single administration of MNPs at concentration of 3000 mg kg^−1^ led to a decreased physical activity, the predominance of sleep periods, lethargy, and reduced acts of grooming in mice during first 10 h after injection. Later, the behavior of the animals did not differ from that in the control groups, except for the acts of grooming, which continued to be reduced. As a result, the state of the coat and skin was changed: the signs of hypersalivation were found on abdominal surface. Intermediate and minimal doses of cobalt ferrite MNPs did not affect the mice behavior notably (see Tables S3–S8).

All studied doses of MNPs caused impaired motor activity, “dragging” of the hind limbs during movement, spasm of the abdominal muscles, the predominance of sleep periods, and lethargy in rats during 10 h after administration. These signs were most pronounced immediately after the MNPs injection. Moreover, mild response to stimuli as well as reduced feed/water intake were noted within the first 3 h after MNPs administration. However, a day later and further (within 30 days of observation), described behavioral disturbances did not appear in all experimental groups of rats (see Tables S9–S14).

#### 3.2.3. MNPs Effect on Blood Cells

The examination of MNPs long-term dynamic impact on the blood cells was carried out on the days 7, 14, and 30 after injection. First, a blood drop smear obtained from the tail vein of the mice was examined. No differences were detected on days 7–30 for all investigated doses. The study of peripheral blood smears of male and female rats injected with MNPs at concentration 600 and 1384.6 mg kg^−1^ revealed accumulation of red blood cells (RBC) with pathologically altered forms (echinocytes) in some fields of view (Figure S4). Minimal dose of MNPs did not cause any changes in RBC morphology.

The quantitative analysis of mice blood cells was carried out on day 30 after MNPs injection. The obtained data on the MNPs effect on the leukocyte formula are presented in Supplementary Tables S15 and S16. The analysis of these results indicates statistically insignificant differences in blood leukocyte composition of male and female mice compared with control groups. The data of MNPs impact on experimental rats leukocyte formula are presented in Supplementary Tables S17 and S18. Analysis of the obtained results indicates a statistically significant increase in the number of monocytes in female rats, which were administered the dose of 1384.6 mg kg^−1^ MNPs, compared to the intact and vehicle groups. For other indicators, no differences were found in blood leukocyte composition of male and female rats in comparison with control groups. 

Thus, we can conclude that minimal and intermediate doses of MNPs do not affect blood cells of both male and female mice and rats. However, the maximum dose results in monocytes number increase in female rats as well as in rats red blood cells morphology changes. 

#### 3.2.4. Blood Biochemical Parameters of Laboratory Animals in the Study of MNPs Acute Toxicity

MNPs injection at a dose of 230 mg kg^−1^ as well as 1300 mg kg^−1^ to male mice caused 25% increase in the amount of total protein (p_K-W_ < 0.05, Kruskal–Wallis test) (see Table 1 and Table S19). In female mice, the total protein was reduced after administration of MNPs at concentration 3000 mg kg^−1^. Moreover, injection of 1300 and 3000 mg kg^−1^ MNPs to female mice resulted in a decrease of glucose level (see Table 1 and Table S20). Changes in the levels of bilirubin, cholesterol, creatinine, alanine amino-transferase (ALT), aspartate amino-transferase (AST), and gamma-glutamyl transferase (GGT) at all investigated concentrations of MNPs were not revealed.

The blood biochemical parameters of male and female rats after MNPs injection, which demonstrated statistically significant changes compared to control animals, are presented in Table 2, Tables S21 and S22. Other investigated biochemical parameters did not differ from the control ones.

Thus, the blood biochemical parameters of experimental animals allow to conclude about the non-dose-dependent effect of the investigated MNPs on an increase in glucose level, and on a decrease in bilirubin, ALT, and AST. Evaluation of the results for other indicators does not allow to unambiguously conclude about the functional state of animals’ systems and organs after MNPs single administration—for this assessment, microscopic description of the internal organs state should be carried out.

#### 3.2.5. Histological Studies in Acute Toxicity

After 30 days of cobalt ferrite MNPs i.p. injection, the following organs of the animals underwent histological examination: lymph node, spleen, liver, kidney, stomach, intestines, heart, lungs, brain, testis, and ovaries. These organs are usually analyzed in the in vivo studies of MNPs toxicity [29,30]. Similar results were obtained for mice and rats. The data of mice organs study are presented.

Lymph node. It was found that all investigated doses of MNPs do not cause changes in lymph node structure. It must be noted, that after MNPs injection at maximum or intermediate dose, nanoparticles were found in the inter-nodular zones, the paracortical zone, and the medulla in the presence of impregnation. Minimal dose of MNPs resulted in insignificant impregnation of lymph nodes. Wherein, MNPs were distributed mainly intracellularly or along the sinus system in all cases (Figure S5). 

Spleen. All studied doses of MNPs did not affect animal spleen—the organ structure was not changed. Only the cells of the organ connective tissue capsule were impregnated with MNPs. Intraorganic MNPs were not detected (Figure S6).

Testis. Pronounced organ changes in the testis and epididymis were not determined after MNPs injection at concentrations of 230 and 3000 mg kg^−1^ for mice as well as 106.2 and 600 mg kg^−1^ for rats. In the convoluted tubules of the testis, spermatozoa were found at different stages of spermatogenesis. The undulation of spermatogenesis was discernible. The epididymis canal was lined with multi-row epithelium. No signs of impregnation with dark particles were found. However, after MNPs administration at dose of 1300 mg kg^−1^ for mice as well as 1384.6 mg kg^−1^ for rats it was revealed that the connective tissue structures of the vaginal membrane of the testis contained numerous cells impregnated with dark particles. No MNPs were found in intraorgan structures. The convoluted seminiferous tubules had a preserved shape and size, but signs of undulation of spermatogenesis were not detected. In a number of tubule sections, signs of spermatogenic cell prolapse and “baldness” of spermatogenic epithelium were found. There were practically no spermatozoa at the final stages of spermatogenesis (Figure S7). Probably, such a difference in MNPs doses effect can be directly related to the process of MNPs i.p. injection.

Injection site. Connective tissue structures were clearly impregnated with dark particles after injection of MNPs at all doses. MNPs were located mainly intracellularly. In the muscle tissue, MNPs lied in the intercellular substance, without penetrating the muscle fibers (Figure S8).

All other listed organs that were examined after MNPs administration did not demonstrate any structural changes and impregnation with MNPs (Figures S9–S16). Thus, histological examination did not reveal pathological changes in most of the studied organs. The expansion and plethora of blood vessels, determined in some organs, may be a manifestation of reactive reversible changes. The lymphoid organs and connective tissue structures were found to be impregnated with MNPs, mainly intracellularly. 

### 3.3. Magnetic Properties of MNPs after Intratumoral Administration

**Figure 3:**
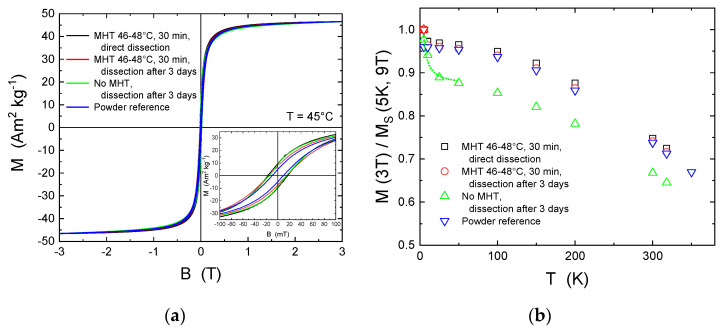
Magnetic properties of MNPs after i.t. injection. (**a**) Magnetic hysteresis of CoFe_2_O_4_ MNPs in tumor tissue after MHT (direct dissection and after 3 days), without MHT, and the powder reference; (**b**) normalized magnetization of the four samples as function of temperature.

Since cobalt ferrite MNPs do not result in instantaneous toxic effects after injection and long-term effects at typical MHT concentrations seem acceptable for small animals, we further investigated the stability of MNPs after i.t. administration and MHT. Figure 3a presents magnetic hysteresis loops of MNPs in tumor tissue and the powder reference at T = 45 °C, i.e., the typical temperature at which MHT is applied. It must be noted that room temperature data are almost identical [20]. Here, we compared MNPs in tumor tissue cut from the darkest spot (highest MNPs concentration) from the following samples: (1) After one cycle of MHT at 46–48 °C for 30 min, directly dissected; (2) with and without the identical MHT treatment but dissected after 3 days; and (3) the MNPs powder reference sample. Since the total mass of MNPs was only known for the reference powder, thus the MNPs bearing tumor tissue samples were scaled on this reference. All samples demonstrated nearly identical magnetic response. As shown in the inset of Figure 3a, however, the reference sample had a slightly smaller coercive field and remanent magnetization. This is likely due to enhanced dipole–dipole interactions of the MNPs in tumor tissue. The similarity of the hysteresis loops demonstrates that the MNPs should be considered as rather stable in organism, at least for 3 days.

Figure 3b presents the temperature dependence of the magnetization M at B = 3 T normalized to M(5 K, 9 T) for all four samples. Such scaling allows to address the degradation of MNPs within tumor tissue. While for the reference powder the ratio M(3T)/M(9T) = 0.96 at T = 5 K accounts for spin disorder often observed at MNPs surfaces, the MHT-treated samples show similar ratios within 1%. This means that the MNPs are highly unaffected in living cells environment after MHT. The very similar temperature dependence of samples after MHT and the reference powder underlines this further.

Interestingly, the injected MNPs dissected after 3 days but without MHT behaved differently. We obtained a steep decrease from 5 to 25–50 K before this sample resembles the M(T) shape of the others at lower level. Such sharp decrease originates from a highly enhanced relative portion of paramagnetic ions, which follows a hyperbolic function obeying the Curie law as indicated by the green dotted line. With the rough estimates that the saturation magnetization of MNPs is not different from the others we find a relative reduction of about 10% for all temperatures. Assuming further that the magnetization of CoFe_2_O_4_ is 2.4 µ_B_ per formula unit and the magnetization of free Co^2+^ and Fe^3+^ ions is 4.8 µ_B_ and 5.9 µ_B_ per ion (thus 1 × 4.8 + 2 × 4.8 µ_B_ per formula unit), respectively, we find that about 2–3% of MNPs mass were dissolved into ions within 3 days inside the tumor. Most importantly, such feature was not observed after MHT. The results suggest that MHT inactivates such dissolution after a single treatment of 30 min at 46–48 °C within the following 3 days which might be associated with the death of the respective malignant cells bearing the MNPs.

In the light of such small changes of the MNPs magnetic properties for the four investigated samples, the MNPs should enable efficient MHT for at least 3 days in the tumor. Figure S17 presents the minor magnetic hysteresis loops in B = ± 30 mT at T = 45 °C. All loops are normalized to the magnetization at 30 mT. Indeed, we cannot obtain significant differences.

## 4. Discussion

MNPs can provide local and, most importantly, controlled heating of the tumor that makes MHT a promising approach for the treatment of malignant neoplasms. The main criteria for choosing perspective MNPs for MHT are their magnetic properties and biocompatibility. Ferrite nanoparticles incorporated with Co^2+^ ions are actively investigated due to their enhanced response to AMF [31]. Previously, we demonstrated that 12 nm citrate-functionalized CoFe_2_O_4_ MNPs have high SLP values and provide controlled tumor heating at three different temperatures (43 °C, 48 °C, and 60 °C), which results in 100% effectiveness of non-metastatic murine tumor therapy and up to 40% for metastatic tumor [20]. However, the effect of MNPs on animals, as well as the ability of MNPs to maintain magnetic properties throughout all days of MHT after i.t. injection was not investigated in detail. Thus, in the current paper, we continued the precise study of synthesized MNPs in the physiological environment. First, the research of their possible toxicity was carried out. Most of the studies are devoted to the cobalt ferrite MNPs biocompatibility investigation after i.v. administration [19,32]. However, the used concentrations of MNPs are often not sufficient to achieve the required temperatures for MHT. Therefore, for MHT the most effective way to inject MNPs is i.t. administration [33]. Wherein, the tolerable dose of MNPs is preliminarily determined based on the toxicological results for MNPs due to i.p. injection [28]. To the best of our knowledge such studies for cobalt ferrite MNPs are absent. 

In this study, we revealed that single administration of described MNPs in doses close to or even higher than that used for MHT (minimal and intermediate doses), has no significant effect on the general condition of laboratory animals, as well as on their leukocyte formula. Only the maximum investigated dose of MNPs had an impact on the following indicators of animals: (1) survival, (2) body weight decrease in male mice, (3) pronounced presence of RBC with pathologically altered forms in rats, and (4) increase in the number of monocytes in female rats. However, by the 30th day, the weight of the mice returned to the normal one. Thus, it is not worth judging the toxic effect of MNPs on the basis of this indicator. The presence of erythrocytes “echinocyte forms” indicates the change in average membrane curvature caused by cells interaction with MNPs, which results in the distraction of lipids away from the RBC membrane [34]. Elevated level of monocytes is usually due to infections, blood disorders, or an increased immunogenic response. It is known that rodents have different innate/adaptive immune responses depending on their gender [35]. Female mice and rats demonstrate stronger phagocytic activity of macrophages than males. However, since such an effect from the injected MNPs was observed only in female rats, this may be due to the individual characteristics of the selected animals. Thus, an unambiguous conclusion about the negative effect of a MNPs maximum dose can be made only on the basis of their effect on RBC and animals survival. Further analysis of blood biochemical parameters demonstrated the non-dose-dependent and contradictory effect of MNPs on total protein and glucose level in animals’ blood. Despite the fact that the differences in these indicators were statistically significant, they did not differ much from those in the control animals. An observed statistically significant decrease in ALT and AST, as well as bilirubin in rats’ blood was also within the normal range for this type of animals. Moreover, it is not considered a diagnostic criterion. On the contrary, liver dysfunction is accompanied by an increase in these indicators [36]. Finally, lowered blood cholesterol level found in rats after MNPs administration can be caused by liver disease. Since MNPs impact on liver and kidney is the most common side effect for a large number of MNPs [37,38], histological study of these organs after cobalt ferrite MNPs injection was carried out and demonstrated no visible changes in their structure even in case of tissue impregnation with MNPs. Moreover, MNPs did not affect lymph nodes, spleen, stomach, intestines, heart, lungs, and brain of experimental animals. The observed negative impact of MNPs at intermediate and maximum doses on the testis of male mice and rats, respectively, against the background of MNPs effect absence on ovaries in females is associated with MNPs presence in testis. Thus, we can conclude that investigated MNPs do not have toxic effect on reproductive system as well regardless of the animals’ sex. No difference in the effect of nanoparticles on the testis and ovaries of mice was also shown in the study of Chen et al. [39]. Altogether, these data indicate that MNPs, i.p. injected at minimal dose, do not have severe toxic effects on animals. Intermediate dose of MNPs affects certain indicators of blood, but the effect is found only in rats. Therefore, this can be due to individual sensitivity of the organism to the drug. At the same time MNPs at maximum dose demonstrated toxic effects for both types of animals. 

It should be noted once more, that MNPs effect on male animals was more pronounced compared to the female ones in general. Males showed greater lethality after MNPs injection, a significant decrease in body weight, and impaired spermatogenesis in case of organ impregnation with MNPs. Such a difference in the response of two animals’ sexes to external impact is quite understandable. Back in the 1990s the biomedical research community demonstrated the importance of sex as a biological variable in laboratory and clinical experiments [35]. This fact emphasizes the importance of this study carried out on both sexes of two animal species. 

The investigations of the magnetic properties of MNPs directly after MHT and after 3 days suggest a good stability of CoFe_2_O_4_ in the organism and no degradation effects as compared to the reference powder can be detected. Thus, due to their high magnetization and huge magnetic anisotropy, cobalt ferrite MNPs are able to provide effective tumor heating at a dose that does not have a toxic effect on the organism. 

As compared to CoFe_2_O_4_, iron oxide (Fe_3_O_4_) MNPs are widely investigated and exploited for MHT [2,5]. However, besides described problems with aggregation and oxidation they demonstrate toxic effect on hepatic and renal tissues (oxidative cell damage) after i.p. injection at high concentrations [40]. Wherein, a negative impact is shown starting with total MNPs concentration in the mouse organism of about 140–280 mg kg^−1^ (20–40 mg kg^−1^ daily for 1 week), i.e., less than the doses we investigated for cobalt ferrite MNPs. Together with the fact, that in order to increase the stability and biocompatibility of iron oxide MNPs, it is necessary to additionally modify their surface [41], which often leads to deteriorated magnetic properties. Thus, the investigated CoFe_2_O_4_ MNPs are perspective materials for tumor treatment by MHT. Moreover, we describe citrate-functionalized cobalt ferrite MNPs and propose to use them for MHT. Previously Akhtar et al. also demonstrated that such MNPs have less toxicity to the body compared to bare or PEG-coated CoFe_2_O_4_ MNPs [42]. 

Finally, not only the chemical composition of MNPs determines their biocompatibility and heating effectiveness, but also MNPs shape and size. It was shown that depending on shape (spheres, cubes, rods, flowers, discs, etc.,) iron oxide MNPs demonstrate different SLP values [2,43]. For example, if we compare iron oxide MNPs of similar magnetic volumes but various shapes, their effectiveness for MHT (SLP) decreases in the following order: nanorods–cubes–spheres. However, it should be considered that rod MNPs are commonly more toxic for cells [44]. For cobalt ferrite MNPs such data on the impact of shape on their biocompatibility and heating efficiency are less numerous. Moreover, recently it was demonstrated that the optimal size of MNPs for effective MHT is ~16 nm for Fe_3_O_4_ and ~6 nm for CoFe_2_O_4_ [45]. Nevertheless, these results are based only on theoretical calculations. Thus, further studies of cobalt ferrite MNPs heating efficiency in combination with their toxicity depending on shape and size will allow to develop the most promising platform for MHT application. 

## Figures and Tables

**Table 1:** Summary of mice reliable biochemical parameters changes (in the study of acute toxicity), median values are presented. * *p* < 0.05 (Kruskal-Wallis One-way ANOVA test).

0 mg kg^−1^	230 mg kg^−1^	1300 mg kg^−1^	3000 mg kg^−1^
**Total Protein (g L^−1^)-Male/Female**			
68.9/87.2	86.5 */87.5	85.75 */88.65	69.6/64.7 *
**Venous blood plasma glucose (mM L^−1^)-Female**			
9.21	6.84	6.01 *	5.56 *

**Table 2:** Summary table of rats’ reliable biochemical parameters changes (in the study of acute toxicity), median values are presented. * *p* < 0.05 (Kruskal–Wallis one-way ANOVA test).

0 mg kg^−1^	106.2 mg kg^−1^	600 mg kg^−1^	1384.6 mg kg^−1^
**Venous Blood Plasma Glucose (mM L^−1^)-Male/Female**			
7.55/7.59	7.29/7.95	9.38 */8.91	10.43 */11.6 *
**Total bilirubin (mM L^−1^)-Male/Female**			
7.8/7.2	6.6/6.6	1.45 */0.8 *	1.3 */2.45 *
**Total cholesterol (mM L^−1^)-Male/Female**			
1.81/1.76	1.89/1.59 *	1.24 */1.37	1.47 */1.65
**ALT (U L^−1^)-Male/Female**			
88.75/75.19	77.3/58.87	53.86 */50.71 *	58.44 */62.13
**AST (U L^−1^)-Male/Female**			
144.1/149.78	132.9/132.21 *	137.22/146.73	95.09 */94.05 *

## Data Availability

The datasets generated during the current work are available from the corresponding author on reasonable request.

## Supplementary Materials

The following are available online at https://www.mdpi.com/article/10.3390/nano12010038/s1, Figure S1. ^57^Fe-Mössbauer absorption spectra of Co^57^Fe_2_O_4_ MNPs. The spectra of the initial MNPs, MNPs after 3-day and 6-day *in vitro* incubation with RAW 264.7 cells are presented. Red and blue lines are partial sextets corresponding to magnetic sublattices A and B. The small cyan doublet, which is present in all spectra, is explained by the presence of an insignificant fraction of ultrafine MNPs, Figure S2. Comparative analysis of male and female mice number (%) with negative body weight gain in the following groups: (a) 3000 mg kg^−1^ (p_k-w_ < 0.05); (b) 1300 mg kg^−1^ (p_k-w_ < 0.05); (c) 230 mg kg^−1^ (p_k-w_ > 0.05); (d) vehicle (p_k-w_ > 0.05). Kruskal-Wallis One-way ANOVA test, Figure S3. Comparative analysis of male and female rats number (%) with negative body weight gain in the following groups: (a) 1384.6 mg kg^−1^ (p_k-w_ > 0.05); (b) 600 mg kg^−1^ (p_k-w_ > 0.05); (c) 106.2 mg kg^−1^ (p_k-w_ > 0.05); (d) vehicle (p_k-w_ > 0.05). Kruskal-Wallis One-way ANOVA test, Figure S4. Micrographs of rats peripheral blood smears 30 days after MNPs acute toxicity experiment start. (a) Blood cells of female rat from intact group—type and shape of red blood cells (RBC) are without pathological changes; (b) Blood cells of female rat injected with 600 mg kg^−1^ cobalt ferrite MNPs—pathological forms of erythrocytes (echinocytes) are observed (indicated by arrows); (c) Blood cells of female rat injected with 1384.6 mg kg^−1^ cobalt ferrite MNPs—pathological forms of erythrocytes (echinocytes) are observed (indicated by arrows), Figure S5. Micrographs of mice lymph node after MNPs injection. (a) Administration of MNPs at minimal dose. Cortex of lymph node. A site of a lymphoid nodule with a reactive center. Cells impregnated with MNPs (dark) in the sinus system; (b) Injection of MNPs at intermediat dose. Internodal distribution of impregnated cells. The impregnated cells are large, polygonal in shape, their nuclei are free of MNPs; (c) Administration of MNPs at maximum dose. Cortex and medulla. Slight impregnation of extraorganic connective tissue with MNPs. Hematoxylin-eosin staining. No visible changes in lymph node structure are found after MNPs injection at all tested doses: cortex and medulla of this organ are distinguishable; the reactive centers of lymphoid nodules as well as immunoblasts in the lymphoid tissue of the brain cords are clearly defined, Figure S6. Micrographs of mice spleen after MNPs injection. (a) Administration of MNPs at minimal dose. White and red spleen pulp. The connective tissue structure located outside the organ is impregnated with MNPs (dark); (b) Injection of MNPs at intermediat dose. Impregnation of the connective tissue capsule cells with MNPs. Megakaryocytes in the red pulp; (c) Administration of MNPs at maximum dose. Impregnation of the connective tissue capsule cells with MNPs. The red pulp contains a large number of megakaryocytic cells. Hematoxylin-eosin staining. The organ structure is not changed: the structures of the white pulp, such as periarterial lymphoid sheaths and lymphoid nodules, are distinguishable. The red pulp is abundantly supplied with blood. Signs of antigen-independent hematopoiesis (megakaryocytes in the red pulp) and immunopoiesis are distinguishable. The reactive centers of the lymphoid nodules are clearly defined, Figure S7. Micrographs of mice testis after MNPs injection. (a) Administration of MNPs at minimal dose; (b) Injection of MNPs at intermediat dose; (c) Administration of MNPs at maximum dose. Insertion demonstrates the convoluted tubules of the testis, in which spermatogonia, spermatocytes, and spermatids are clearly differentiated. Hematoxylin-eosin staining, Figure S8. Micrographs of mice injection site after MNPs administration. (a) Administration of MNPs at minimal dose; (b) Injection of MNPs at intermediat dose; (c) Administration of MNPs at maximum dose. Connective tissue structures are clearly impregnated with dark particles. Insertion in (a) demonstrates impregnated connective tissue cells. Hematoxylin-eosin staining, Figure S9. Micrographs of mice liver after MNPs injection. (a) Administration of MNPs at minimal dose. Lobular organization of the parenchyma. Radial arrangement of hepatocytes beams; (b) Injection of MNPs at intermediat dose. Hepatocytes are without pronounced changes, have 1–2 nuclei, form hepatic tracts; (c) Administration of MNPs at maximum dose. There are small round-cell infiltrates inside the lobules, single cells are impregnated with MNPs (arrow and insertion). Hematoxylin-eosin staining. The organ-specific structure of the liver is not changed due to MNPs administration at all investigated doses: lobularity of the parenchyma is expressed. The hepatocyte beams are oriented radially from the central vein. Hepatocytes containe 1–2 nuclei and oxyphilically stained cytoplasm. Portal zones containe small round-cell infiltrates, while single cells are impregnated with MNPs, Figure S10: Micrographs of mice kidneys after MNPs injection. (a) Administration of MNPs at minimal dose; (b) Injection of MNPs at intermediat dose; (c) Administration of MNPs at maximum dose. Hematoxylin-eosin staining. No organ changes are found after animals’ injection with MNPs at all tested concentrations: the structure is clearly subdivided into cortex and medulla. Arc vessels are full-blooded. The renal corpuscles include the vascular glomerulus. The cavity of the renal corpuscle is not expanded. The convoluted tubules of the cortex are lined with prismatic epithelium without visible changes. The renal tubules of the medulla are without visible changes, Figure S11: Micrographs of mice stomach after MNPs injection. (a) Administration of MNPs at minimal dose; (b) Injection of MNPs at intermediat dose; (c) Administration of MNPs at maximum dose. Layered organization of the mucosa, submucosa, muscular, and serous membranes. Insertion in (c) demonstrates the MNPs impregnation of mesentery connective tissue structures. Hematoxylin-eosin staining. None of the MNPs doses lead to significant changes in organ morphology: the layered organization of the mucosa, submucosa, muscle, and serous membranes is preserved. The gastric pits are not deep, lined with prismatic epithelium. The proper glands of the mucous membrane containe numerous parietal cells, in the bottom parts—small, basophilic stained cells. The lamina propria of the mucous membrane in the region of the bottom parts of the glands containe numerous full-blooded vessels. Serous membrane is unchanged, Figure S12: Micrographs of mice intestines after MNPs injection. (a) Administration of MNPs at minimal dose; (b) Injection of MNPs at intermediat dose; (c) Administration of MNPs at maximum dose. Hematoxylin-eosin staining. The structure of the intestines is not changed due to MNPs administration: the intestinal wall is formed by mucosa, submucosa, muscular, and serous membranes, which have a typical layer-by-layer organization. Folds and crypts are found in the relief. The epithelial lamina of the mucosa containe numerous goblet cells, Figure S13: Micrographs of mice heart after MNPs injection. (a) Administration of MNPs at minimal dose; (b) Injection of MNPs at intermediat dose; (c) Administration of MNPs at maximum dose. Myocardium: working cardiomyocytes. Hematoxylin-eosin staining. No visible changes in the structure of the heart are found after MNPs injection: the myocardium is represented by functional fibers formed by working cardiomyocytes. Between the cardiomyocytes, there are thin layers of connective tissue, which are supplied with blood by small blood capillaries of the somatic type, Figure S14: Micrographs of mice lung after MNPs injection. (a) Administration of MNPs at minimal dose; (b) Injection of MNPs at intermediat dose; (c) Administration of MNPs at maximum dose. Broncho-associated lymphoid tissue is visualized in the wall of the bronchi. Hematoxylin-eosin staining. No visible changes are found in the respiratory section due to MNPs administration at all investigated doses: bronchi of various sizes are determined in the sections of the lungs. In the middle bronchi, mucosa, submucosa, fibrocartilaginous and adventitious membranes are differentiated. In the mucous and submucosal membranes, lymphoid tissue is visualized, located diffusely or in the form of lymphoid nodules, Figure S15: Micrographs of mice brain after MNPs injection at intermediat dose. The identical results are obtained for other doses. (a) The neocortex is of the sensorimotor type. Molecular, outer and inner granular layers are clearly expressed. The pyramidal layers are poorly expressed. White matter. (b) Hippocampus. Neuroarchitectonics has a characteristic organization. Hematoxylin-eosin staining. The studied parts of the brain are not changed: the neocortex has a distinct layered organization. Neural populations characteristic of 1–6 cortex layers have typical localization. Diffuse or focal changes are not identified. The hippocampus has a characteristic structure, neuro- and glioarchitectonics are without features. The pia mater is unchanged, Figure S16: Micrographs of mice ovaries after MNPs injection. (a) Administration of MNPs at minimal dose; (b) Injection of MNPs at intermediat dose; (c) Administration of MNPs at maximum dose. Hematoxylin-eosin staining. In the cortex, primordial, primary, secondary, mature follicles, atretic formations, and yellow bodies are found. Secondary follicles containe developed follicular epithelium. The ovocyte in them is surrounded by a zona pellucida. Two layers of theca are differentiated. Primordial follicles are few in number. Atretic follicles are found in large numbers in the deep layers of the cortex. The vessels of the medulla are dilated, filled with blood. Oviduct wall is without visible changes, Figure S17. Minor magnetic hysteresis in B = ± 30 mT at T = 45 °C. All loops are normalized to the magnetization at 30 mT, Table S1. Evaluation of animal survival in the study of cobalt ferrite MNPs acute toxicity (i.p. injection), Table S2. Norms of intact animals (mice and rats) general condition, Table S3. Indicators of the mice (males and females) state during the first day after cobalt ferrite MNPs administration at a dose of 3000 mg kg^−1^ in the study of acute toxicity, Table S4. Indicators of the mice (males and females) state during the 2–30 days after cobalt ferrite MNPs administration at a dose of 3000 mg kg^−1^ in the study of acute toxicity, Table S5. Indicators of the mice (males and females) state during the first day after cobalt ferrite MNPs administration at a dose of 1300 mg kg^−1^ in the study of acute toxicity, Table S6. Indicators of the mice (males and females) state during the 2–30 days after cobalt ferrite MNPs administration at a dose of 1300 mg kg^−1^ in the study of acute toxicity, Table S7. Indicators of the mice (males and females) state during the first day after cobalt ferrite MNPs administration at a dose of 230 mg kg^−1^ in the study of acute toxicity, Table S8. Indicators of the mice (males and females) state during the 2–30 days after cobalt ferrite MNPs administration at a dose of 230 mg kg^−1^ in the study of acute toxicity, Table S9. Indicators of the rats (males and females) state during the first day after cobalt ferrite MNPs administration at a dose of 1384.6 mg kg^−1^ in the study of acute toxicity, Table S10. Indicators of the rats (males and females) state during the 2–30 days after cobalt ferrite MNPs administration at a dose of 1384.6 mg kg^−1^ in the study of acute toxicity, Table S11. Indicators of the rats (males and females) state during the first day after cobalt ferrite MNPs administration at a dose of 600 mg kg^−1^ in the study of acute toxicity, Table S12. Indicators of the rats (males and females) state during the 2–30 days after cobalt ferrite MNPs administration at a dose of 600 mg kg^−1^ in the study of acute toxicity, Table S13. Indicators of the rats (males and females) state during the first day after cobalt ferrite MNPs administration at a dose of 106.2 mg kg^−1^ in the study of acute toxicity, Table S14. Indicators of the rats (males and females) state during the 2–30 days after cobalt ferrite MNPs administration at a dose of 106.2 mg kg^−1^ in the study of acute toxicity, Table S15. Assessment of MNPs effect on the male mice leukocyte blood count (in the study of acute toxicity). Table cell designations: Median/Min÷Max/interquartile percentiles (P_25%_÷P_75%_), Table S16. Assessment of MNPs effect on the female mice leukocyte blood count (in the study of acute toxicity). Table cell designations: Median/Min÷Max/interquartile percentiles (P_25%_÷P_75%_), Table S17. Assessment of MNPs effect on the male rats leukocyte blood count (in the study of acute toxicity). Table cell designations: Median/Min÷Max/interquartile percentiles (P_25%_÷P_75%_), Table S18. Assessment of MNPs effect on the female rats leukocyte blood count (in the study of acute toxicity). Table cell designations: Median/Min÷Max/interquartile percentiles (P_25%_÷P_75%_), Table S19. Assessment of MNPs effect on the male mice blood biochemical parameters (in the study of acute toxicity). Table cell designations: Median/Min÷Max/interquartile percentiles (P_25%_÷P_75%_), Table S20. Assessment of MNPs effect on the female mice blood biochemical parameters (in the study of acute toxicity). Table cell designations: Median/Min÷Max/interquartile percentiles (P_25%_÷P_75%_), Table S21. Assessment of MNPs effect on the male rats blood biochemical parameters (in the study of acute toxicity). Table cell designations: Median/Min÷Max/interquartile percentiles (P_25%_÷P_75%_), Table S22. Assessment of MNPs effect on the female rats blood biochemical parameters (in the study of acute toxicity). Table cell designations: Median/Min÷Max/interquartile percentiles (P_25%_÷P_75%_).
