# Supplementary material for: Cobalt Ferrite Nanoparticles for Tumor Therapy: Effective Heating versus Possible Toxicity
Source: Nanomaterials (Basel). 2021 Dec 23;12(1):38. doi: 10.3390/nano12010038 (PMC8746458; doi:10.3390/nano12010038)
Supplement: Supplementary file 1 [file nanomaterials-12-00038-s001.zip › nanomaterials-1500880-supplementary.pdf]

nanomaterials

*Supplementary information*

# Cobalt ferrite nanoparticles for tumor therapy: effective heating versus possible toxicity

**Anastasiia S. Garanina [1], Alexey A. Nikitin [1], Tatiana O. Abakumova [2], Alevtina S. Semkina [3,4], Alexandra O. Prelovskaya [1], Victor A. Naumenko [4], Alexander S. Erofeev [1,5], Peter V. Gorelkin [6], Alexander G. Majouga [1,5,7], Maxim A. Abakumov [1,3,†] and Ulf Wiedwald [1,8,*,†]**

1 National University of Science and Technology «MISiS», 119049, Moscow, Russia; anastasiacit@gmail.com (A.S.G.); nikitin.aa93@mail.ru (A.A.N.); sasha.prelove@gmail.com (A.O.P.); erofeev@polly.phys.msu.ru (A.S.E.); alexander.majouga@gmail.com (A.G.M.); abakumov1988@gmail.com (M.A.A.)
2 Skolkovo Institute of Science and Technology, 121205, Moscow, Russia; sandalovato@gmail.com
3 Department of Medical Nanobiotechnology, Russian National Research Medical University, 117997, Moscow, Russia; alevtina.semkina@gmail.com
4 V. Serbsky National Medical Research Center for Psychiatry and Narcology, 119034, Moscow, Russia; naumenko.vict@gmail.com
5 Department of Chemistry, Lomonosov Moscow State University, 119991, Moscow, Russia
6 Medical Nanotechnology LLC, Skolkovo Innovation Center, 121205, Moscow, Russia; peter.gorelkin@gmail.com
7 D. Mendeleev University of Chemical Technology of Russia, 125047, Moscow, Russia
8 Faculty of Physics and Center for Nanointegration Duisburg-Essen, University of Duisburg-Essen, 47057, Duisburg, Germany
* Correspondence: ulf.wiedwald@uni-due.de
† These authors contributed equally to this work.

**Table S1.** Evaluation of animal survival in the study of cobalt ferrite MNPs acute toxicity (i.p. injection).

| Animal species | Animal sex | Doses mg kg$^{-1}$ | Number of animals in the group | Number of animals: Survived Abs. | Survived % | Died Abs. | Died % |
|---|---|---|---|---|---|---|---|
| Mice | Males | 3000 | 5 | 3 | 60 % | 2 | 40 % |
| | | 1300 | 5 | 5 | 100 % | 0 | 0 % |
| | | 230 | 5 | 5 | 100 % | 0 | 0 % |
| | | 0, intact | 5 | 5 | 100 % | 0 | 0 % |
| | | 0, vehicle | 5 | 5 | 100 % | 0 | 0 % |
| | Females | 3000 | 5 | 5 | 100 % | 0 | 0 % |
| | | 1300 | 5 | 5 | 100 % | 0 | 0 % |
| | | 230 | 5 | 5 | 100 % | 0 | 0 % |
| | | 0, intact | 5 | 5 | 100 % | 0 | 0 % |
| | | 0, vehicle | 5 | 5 | 100 % | 0 | 0 % |
| Rats | Males | 1384,6 | 5 | 5 | 100 % | 0 | 0 % |
| | | 600 | 5 | 4 | 80 % | 1 | 20 % |
| | | 106,2 | 5 | 5 | 100 % | 0 | 0 % |
| | | 0, intact | 5 | 5 | 100 % | 0 | 0 % |
| | | 0, vehicle | 5 | 5 | 100 % | 0 | 0 % |
| | Females | 1384,6 | 5 | 4 | 80 % | 1 | 20 % |
| | | 600 | 5 | 5 | 100 % | 0 | 0 % |
| | | 106,2 | 5 | 5 | 100 % | 0 | 0 % |
| | | 0, intact | 5 | 5 | 100 % | 0 | 0 % |
| | | 0, vehicle | 5 | 5 | 100 % | 0 | 0 % |

Abbreviations: Abs. – absolute.

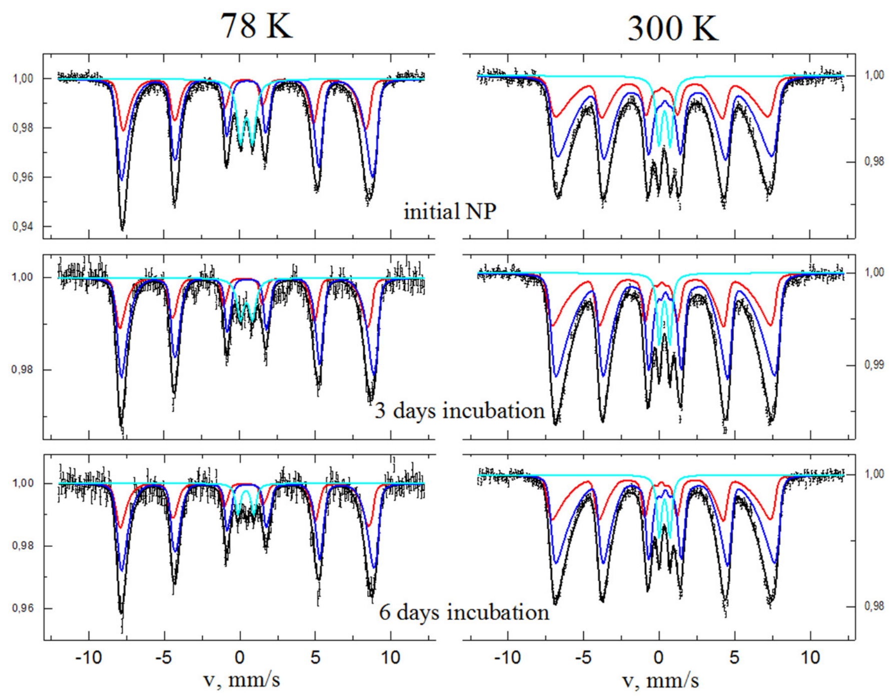


**Figure S1.** $^{57}$Fe-Mössbauer absorption spectra of $Co^{57}Fe_2O_4$ MNPs. The spectra of the initial MNPs, MNPs after 3-day and 6-day *in vitro* incubation with RAW 264.7 cells are presented. Red and blue lines are partial sextets corresponding to magnetic sublattices A and B. The small cyan doublet, which is present in all spectra, is explained by the presence of an insignificant fraction of ultrafine MNPs.

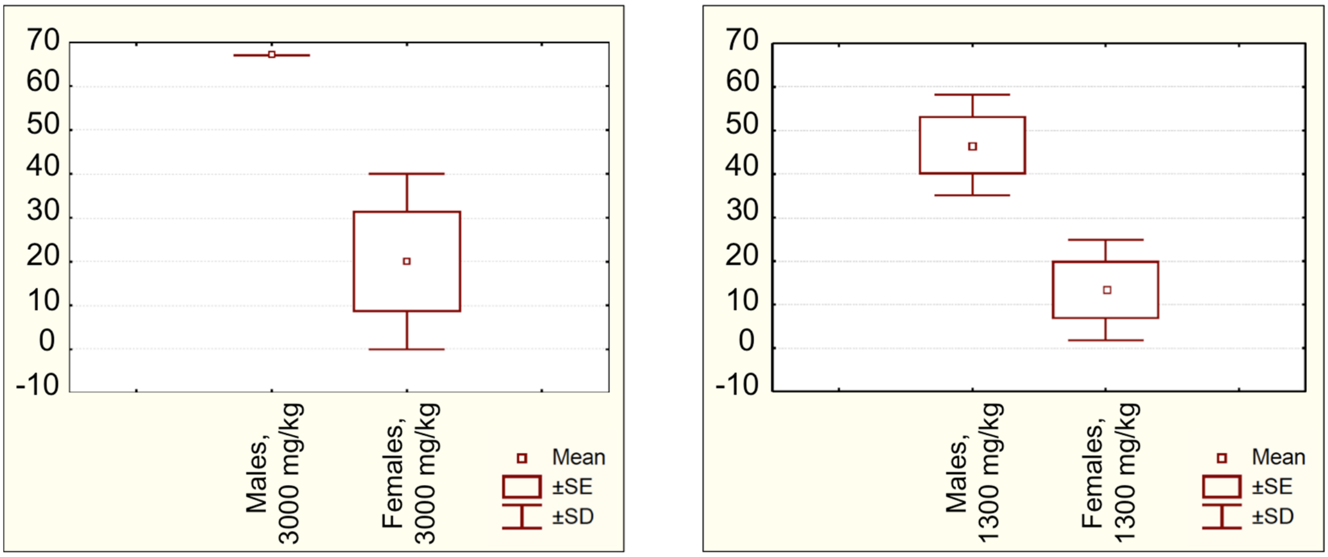


**(a)** **(b)**

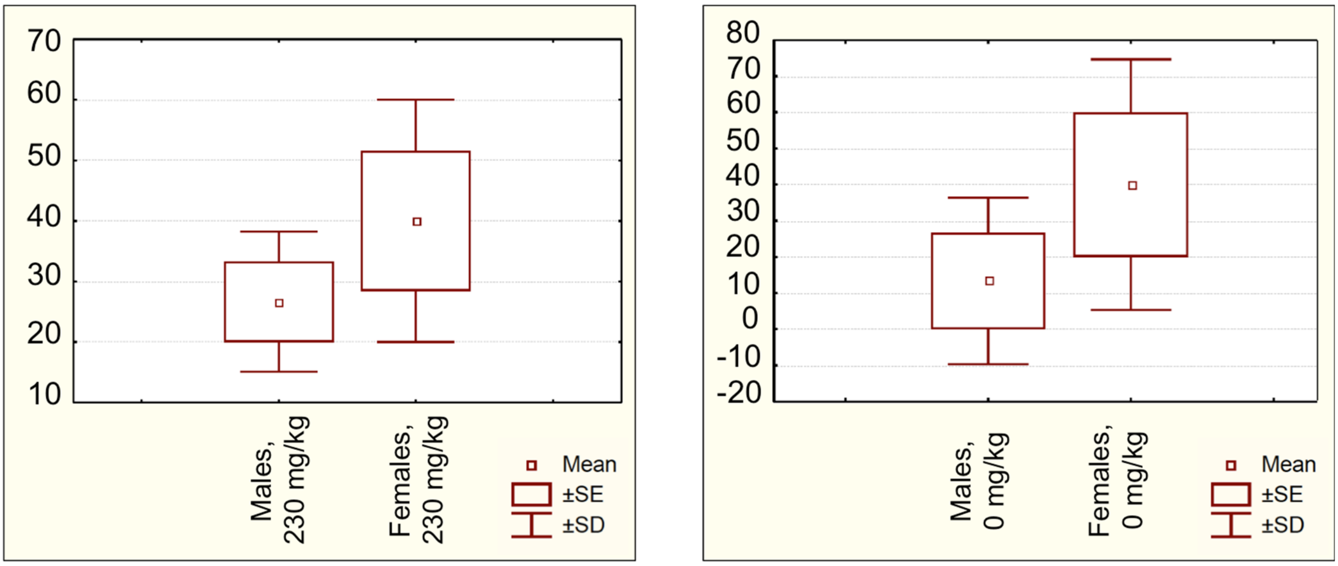


(c) (d)

**Figure S2.** Comparative analysis of male and female mice number (%) with negative body weight gain in the following groups: (**a**) 3000 mg $kg^{-1}$ ($p_{k\text{-}w} < 0{,}05$); (**b**) 1300 mg $kg^{-1}$ ($p_{k\text{-}w} < 0{,}05$); (**c**) 230 mg $kg^{-1}$ ($p_{k\text{-}w} > 0{,}05$); (**d**) vehicle ($p_{k\text{-}w} > 0{,}05$). Kruskal-Wallis One-way ANOVA test.

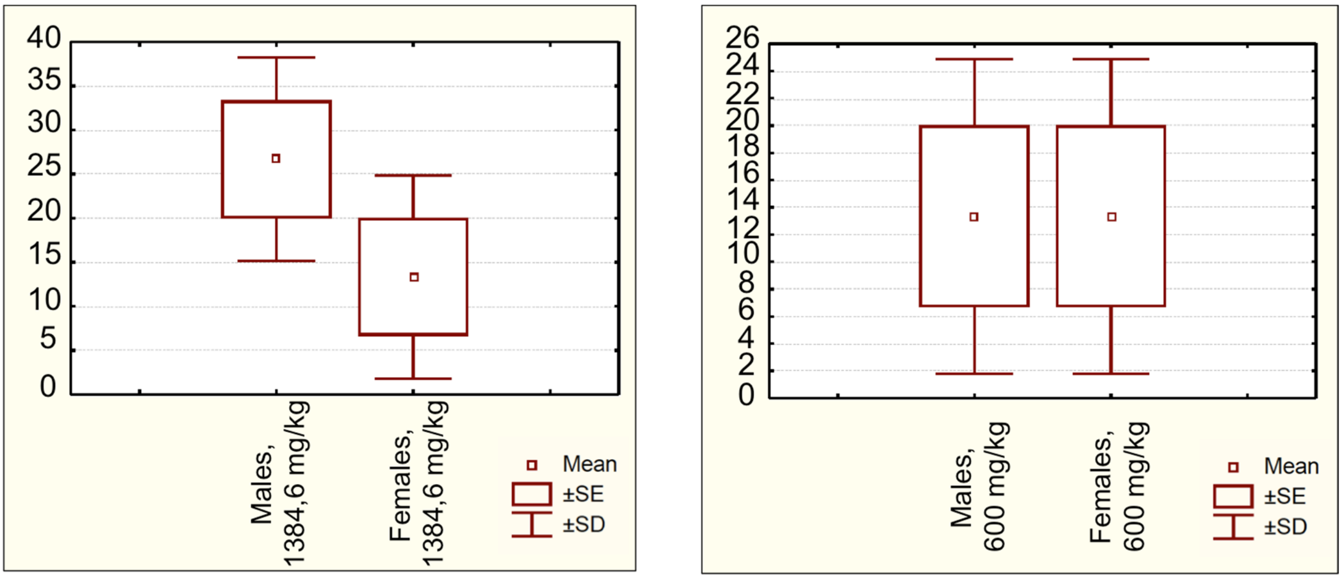


(a) (b)

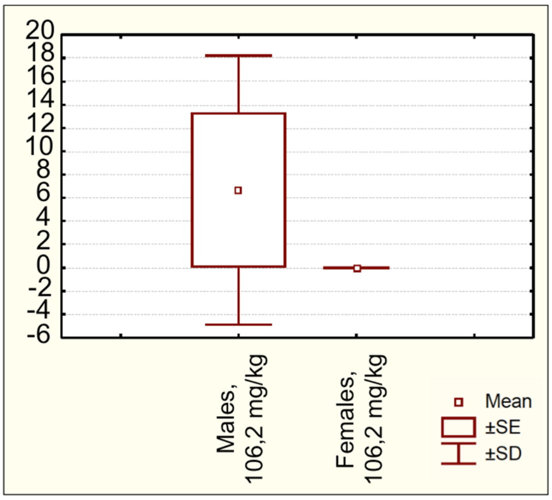


**(c)**

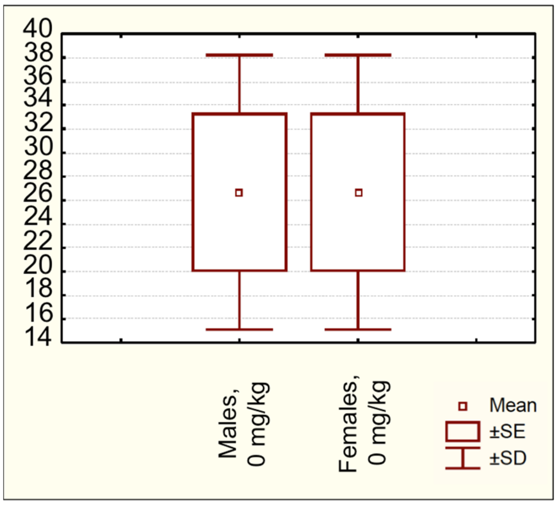


**(d)**

**Figure S3.** Comparative analysis of male and female rats number (%) with negative body weight gain in the following groups: (**a**) 1384,6 mg kg$^{-1}$ ($p_{k\text{-}w}$ > 0,05); (**b**) 600 mg kg$^{-1}$ ($p_{k\text{-}w}$ > 0,05); (**c**) 106,2 mg kg$^{-1}$ ($p_{k\text{-}w}$ > 0,05); (**d**) vehicle ($p_{k\text{-}w}$ > 0,05). Kruskal-Wallis One-way ANOVA test.

**Table S2.** Norms of intact animals (mice and rats) general condition.

| № | Indicator | Description of the norm |
|---|---|---|
| 1 | General condition of animals | Alternation of wakefulness and sleep periods, absence of lethargy, grooming for 10-20 seconds, animals do not have discharge from the eyes, ears, nose, and mouth |
| 2 | Features of behavior | Lack of aggression towards individuals in the group, the animal does not avoid contact with individuals in the group |
| 3 | The intensity and nature of motor activity | There is horizontal and vertical physical activity |
| 4 | The presence and nature of seizures, coordination of movements | Absence of seizures, coordination of movements is not impaired, there is a coordinated work of the front and hind limbs and tail during movement, animals do not make monotonous movements |
| 5 | Skeletal muscle tone | Muscle tone is normal, there is no spontaneous contraction of certain muscle groups, there is no muscle tremor |
| 6 | Reaction to tactile, painful, sound, and light stimuli | There is an immediate reaction of the animal to tactile, pain, sound, and light stimulation |
| 7 | Respiratory rate | 90-160 breaths per minute (on the average) |
| 8 | Heartbeat rhythm | 300-600 beats per minute (on the average) |
| 9 | Condition of the coat and skin | The coat is clean (white), smooth, shiny, uniform, the skin is dry, without signs of hypersalivation or flaking, the skin color is pale, there is no cyanosis or redness of the skin or its areas, the skin is intact, there are no wound foci and foci of necrosis |
| 10 | Coloring of mucous membranes | Pink or pale pink |
| 11 | Pupil size | There is a pupillary reflex, the pupils are not expanded and not narrowed |
| 12 | Tail position | Passive at rest and during rectilinear movement on a flat horizontal surface; when turning the body in a horizontal surface, the tail is used as a balancer; during an approximate vertical stance, the tail is used as a support |
| 13 | The amount and consistency of fecal matter | The number of defecation acts during wakefulness is 3±1 per minute, the consistency is soft, mushy, the color is dark brown |
| 14 | Urinary frequency and urine coloration | The number of urination acts during wakefulness is 1±0.12 in 5-20 minutes, urine color is light yellow |
| 15 | Feed and water consumption | 5 g of granulated SPF compound feed per mouse and 20-25 g per rat per day, animals willingly eat feed, water consumption is without restriction of access |

**Table S3.** Indicators of the mice (males and females) state during the first day after cobalt ferrite MNPs administration at a dose of 3000 mg kg$^{-1}$ in the study of acute toxicity.

| № | Indicator | Watching hours | | | | | | | | | |
|---|---|---|---|---|---|---|---|---|---|---|---|
| | | 1 | 2 | 3 | 4 | 5 | 6 | 7 | 8 | 10 | 24 |
| 1 | General condition of animals | The predominance of sleep periods and lethargy, grooming (rarely) for 3-5 seconds | | | | | | | | | N |
| 2 | Features of behavior | N | N | N | N | N | N | N | N | N | N |
| 3 | The intensity and nature of motor activity | Reduced motor activity | | | | | | | | | N |
| 4 | The presence and nature of seizures, coordination of movements | Abs | Abs | Abs | Abs | Abs | Abs | Abs | Abs | Abs | Abs |
| 5 | Skeletal muscle tone | N | N | N | N | N | N | N | N | N | N |
| 6 | Reaction to tactile, painful, sound, and light stimuli | Weak | | | N | N | N | N | N | N | N |
| 7 | Respiratory rate | N | N | N | N | N | N | N | N | N | N |
| 8 | Heartbeat rhythm | N | N | N | N | N | N | N | N | N | N |
| 9 | Condition of the coat and skin | The coat is "tousled", with signs of hypersalivation on abdominal surface | | | | | | | | | |
| 10 | Coloring of mucous membranes | N | N | N | N | N | N | N | N | N | N |
| 11 | Pupil size | N | N | N | N | N | N | N | N | N | N |
| 12 | Tail position | N | N | N | N | N | N | N | N | N | N |
| 13 | The amount and consistency of fecal matter | N | N | N | N | N | N | N | N | N | N |
| 14 | Urinary frequency and urine coloration | N | N | N | N | N | N | N | N | N | N |
| 15 | Feed and water consumption | N | N | N | N | N | N | N | N | N | N |

Abbreviations: Abs – absent; N – norm.

**Table S4.** Indicators of the mice (males and females) state during the 2-30 days after cobalt ferrite MNPs administration at a dose of 3000 mg kg$^{-1}$ in the study of acute toxicity.

| № | Indicator | Watching days | | | | | | |
|---|---|---|---|---|---|---|---|---|
| | | 2 | 3 | 4 | 5 | 6… | 8… | 30 |
| 1 | General condition of animals | Male death on days 2 and 8 | | | | | | |
| 2 | Features of behavior | N | N | N | N | N | N | N |
| 3 | The intensity and nature of motor activity | N | N | N | N | N | N | N |
| 4 | The presence and nature of seizures, coordination of movements | N | N | N | N | N | N | N |
| 5 | Skeletal muscle tone | N | N | N | N | N | N | N |
| 6 | Reaction to tactile, painful, sound, and light stimuli | N | N | N | N | N | N | N |
| 7 | Respiratory rate | N | N | N | N | N | N | N |
| 8 | Heartbeat rhythm | N | N | N | N | N | N | N |
| 9 | Condition of the coat and skin | The coat is "tousled", with signs of hypersalivation on abdominal surface | | | | | | |
| 10 | Coloring of mucous membranes | N | N | N | N | N | N | N |
| 11 | Pupil size | N | N | N | N | N | N | N |
| 12 | Tail position | N | N | N | N | N | N | N |
| 13 | The amount and consistency of fecal matter | N | N | N | N | N | N | N |
| 14 | Urinary frequency and urine coloration | N | N | N | N | N | N | N |
| 15 | Feed and water consumption | N | N | N | N | N | N | N |

Abbreviations: N – norm.

**Table S5.** Indicators of the mice (males and females) state during the first day after cobalt ferrite MNPs administration at a dose of 1300 mg $kg^{-1}$ in the study of acute toxicity.

| № | Indicator | Watching hours | | | | | | | | | |
|---|---|---|---|---|---|---|---|---|---|---|---|
| | | 1 | 2 | 3 | 4 | 5 | 6 | 7 | 8 | 10 | 24 |
| 1 | General condition of animals | N | N | N | N | N | N | N | N | N | N |
| 2 | Features of behavior | N | N | N | N | N | N | N | N | N | N |
| 3 | The intensity and nature of motor activity | N | N | N | N | N | N | N | N | N | N |
| 4 | The presence and nature of seizures, coordination of movements | Abs | Abs | Abs | Abs | Abs | Abs | Abs | Abs | Abs | Abs |
| 5 | Skeletal muscle tone | N | N | N | N | N | N | N | N | N | N |
| 6 | Reaction to tactile, painful, sound, and light stimuli | N | N | N | N | N | N | N | N | N | N |
| 7 | Respiratory rate | N | N | N | N | N | N | N | N | N | N |
| 8 | Heartbeat rhythm | N | N | N | N | N | N | N | N | N | N |
| 9 | Condition of the coat and skin | N | N | N | N | N | N | N | N | N | N |
| 10 | Coloring of mucous membranes | N | N | N | N | N | N | N | N | N | N |
| 11 | Pupil size | N | N | N | N | N | N | N | N | N | N |
| 12 | Tail position | N | N | N | N | N | N | N | N | N | N |
| 13 | The amount and consistency of fecal matter | N | N | N | N | N | N | N | N | N | N |
| 14 | Urinary frequency and urine coloration | N | N | N | N | N | N | N | N | N | N |
| 15 | Feed and water consumption | N | N | N | N | N | N | N | N | N | N |

Abbreviations: Abs – absent; N – norm.

**Table S6.** Indicators of the mice (males and females) state during the 2-30 days after cobalt ferrite MNPs administration at a dose of 1300 mg $kg^{-1}$ in the study of acute toxicity.

| № | Indicator | Watching days | | | | | | |
|---|---|---|---|---|---|---|---|---|
| | | 2 | 3 | 4 | 5 | 6… | 8… | 30 |
| 1 | General condition of animals | N | N | N | N | N | N | N |
| 2 | Features of behavior | N | N | N | N | N | N | N |
| 3 | The intensity and nature of motor activity | N | N | N | N | N | N | N |
| 4 | The presence and nature of seizures, coordination of move-ments | N | N | N | N | N | N | N |
| 5 | Skeletal muscle tone | N | N | N | N | N | N | N |
| 6 | Reaction to tactile, painful, sound, and light stimuli | N | N | N | N | N | N | N |
| 7 | Respiratory rate | N | N | N | N | N | N | N |
| 8 | Heartbeat rhythm | N | N | N | N | N | N | N |
| 9 | Condition of the coat and skin | N | N | N | N | N | N | N |
| 10 | Coloring of mucous membranes | N | N | N | N | N | N | N |
| 11 | Pupil size | N | N | N | N | N | N | N |
| 12 | Tail position | N | N | N | N | N | N | N |
| 13 | The amount and consistency of fecal matter | N | N | N | N | N | N | N |
| 14 | Urinary frequency and urine coloration | N | N | N | N | N | N | N |
| 15 | Feed and water consumption | N | N | N | N | N | N | N |

Abbreviations: N – norm.

**Table S7.** Indicators of the mice (males and females) state during the first day after cobalt ferrite MNPs administration at a dose of 230 mg $kg^{-1}$ in the study of acute toxicity.

| № | Indicator | Watching hours | | | | | | | | | |
|---|---|---|---|---|---|---|---|---|---|---|---|
| | | 1 | 2 | 3 | 4 | 5 | 6 | 7 | 8 | 10 | 24 |
| 1 | General condition of animals | N | N | N | N | N | N | N | N | N | N |
| 2 | Features of behavior | N | N | N | N | N | N | N | N | N | N |
| 3 | The intensity and nature of motor activity | N | N | N | N | N | N | N | N | N | N |
| 4 | The presence and nature of seizures, coordination of movements | Abs | Abs | Abs | Abs | Abs | Abs | Abs | Abs | Abs | Abs |

| | | | | | | | | | | | |
|---|---|---|---|---|---|---|---|---|---|---|---|
| 5 | Skeletal muscle tone | N | N | N | N | N | N | N | N | N | N |
| 6 | Reaction to tactile, painful, sound, and light stimuli | N | N | N | N | N | N | N | N | N | N |
| 7 | Respiratory rate | N | N | N | N | N | N | N | N | N | N |
| 8 | Heartbeat rhythm | N | N | N | N | N | N | N | N | N | N |
| 9 | Condition of the coat and skin | N | N | N | N | N | N | N | N | N | N |
| 10 | Coloring of mucous membranes | N | N | N | N | N | N | N | N | N | N |
| 11 | Pupil size | N | N | N | N | N | N | N | N | N | N |
| 12 | Tail position | N | N | N | N | N | N | N | N | N | N |
| 13 | The amount and consistency of fecal matter | N | N | N | N | N | N | N | N | N | N |
| 14 | Urinary frequency and urine coloration | N | N | N | N | N | N | N | N | N | N |
| 15 | Feed and water consumption | N | N | N | N | N | N | N | N | N | N |

Abbreviations: Abs – absent; N – norm.

**Table S8.** Indicators of the mice (males and females) state during the 2-30 days after cobalt ferrite MNPs administration at a dose of 230 mg kg$^{-1}$ in the study of acute toxicity.

| № | Indicator | Watching days | | | | | | |
|---|---|---|---|---|---|---|---|---|
| | | 2 | 3 | 4 | 5 | 6… | 8… | 30 |
| 1 | General condition of animals | N | N | N | N | N | N | N |
| 2 | Features of behavior | N | N | N | N | N | N | N |
| 3 | The intensity and nature of motor activity | N | N | N | N | N | N | N |
| 4 | The presence and nature of seizures, coordination of movements | N | N | N | N | N | N | N |
| 5 | Skeletal muscle tone | N | N | N | N | N | N | N |
| 6 | Reaction to tactile, painful, sound, and light stimuli | N | N | N | N | N | N | N |
| 7 | Respiratory rate | N | N | N | N | N | N | N |
| 8 | Heartbeat rhythm | N | N | N | N | N | N | N |
| 9 | Condition of the coat and skin | N | N | N | N | N | N | N |
| 10 | Coloring of mucous membranes | N | N | N | N | N | N | N |
| 11 | Pupil size | N | N | N | N | N | N | N |
| 12 | Tail position | N | N | N | N | N | N | N |
| 13 | The amount and consistency of fecal matter | N | N | N | N | N | N | N |
| 14 | Urinary frequency and urine coloration | N | N | N | N | N | N | N |
| 15 | Feed and water consumption | N | N | N | N | N | N | N |

Abbreviations: N – norm.

**Table S9.** Indicators of the rats (males and females) state during the first day after cobalt ferrite MNPs administration at a dose of 1384,6 mg kg$^{-1}$ in the study of acute toxicity.

| № | Indicator | Watching hours | | | | | | | | | |
|---|---|---|---|---|---|---|---|---|---|---|---|
| | | 1 | 2 | 3 | 4 | 5 | 6 | 7 | 8 | 10 | 24 |
| 1 | General condition of animals | Movement disturbance, "dragging" of the hind limbs when moving, spasm of the abdominal muscles, the predominance of sleep periods, lethargy | | | | | | | | | N |
| 2 | Features of behavior | N | N | N | N | N | N | N | N | N | N |
| 3 | The intensity and nature of motor activity | Reduced motor activity | | | | | | | | | N |
| 4 | The presence and nature of seizures, coordination of movements | Periodically impaired coordination of movement | | | | | | Abs | Abs | Abs | Abs |
| 5 | Skeletal muscle tone | N | N | N | N | N | N | N | N | N | N |
| 6 | Reaction to tactile, painful, sound, and light stimuli | Weak | | | N | N | N | N | N | N | N |
| 7 | Respiratory rate | N | N | N | N | N | N | N | N | N | N |
| 8 | Heartbeat rhythm | N | N | N | N | N | N | N | N | N | N |
| 9 | Condition of the coat and skin | N | N | N | N | N | N | N | N | N | N |
| 10 | Coloring of mucous membranes | N | N | N | N | N | N | N | N | N | N |
| 11 | Pupil size | N | N | N | N | N | N | N | N | N | N |

| | | | | | | | | | | | |
|---|---|---|---|---|---|---|---|---|---|---|---|
| 12 | Tail position | Periodi-cally vertically up | | N | N | N | N | N | N | N | N |
| 13 | The amount and consistency of fecal matter | N | N | N | N | N | N | N | N | N | N |
| 14 | Urinary frequency and urine coloration | N | N | N | N | N | N | N | N | N | N |
| 15 | Feed and water consumption | Reduced | | N | N | N | N | N | N | N | N |

Abbreviations: Abs – absent; N – norm.

**Table S10.** Indicators of the rats (males and females) state during the 2-30 days after cobalt ferrite MNPs administration at a dose of 1384,6 mg kg$^{-1}$ in the study of acute toxicity.

| № | Indicator | Watching days | | | | | | |
|---|---|---|---|---|---|---|---|---|
| | | 2 | 3 | 4 | 5 | 6… | 16… | 30 |
| 1 | General condition of animals | Female death on day 16 | | | | | | N |
| 2 | Features of behavior | N | N | N | N | N | N | N |
| 3 | The intensity and nature of motor activity | N | N | N | N | N | N | N |
| 4 | The presence and nature of seizures, coordination of movements | N | N | N | N | N | N | N |
| 5 | Skeletal muscle tone | N | N | N | N | N | N | N |
| 6 | Reaction to tactile, painful, sound, and light stimuli | N | N | N | N | N | N | N |
| 7 | Respiratory rate | N | N | N | N | N | N | N |
| 8 | Heartbeat rhythm | N | N | N | N | N | N | N |
| 9 | Condition of the coat and skin | N | N | N | N | N | N | N |
| 10 | Coloring of mucous membranes | N | N | N | N | N | N | N |
| 11 | Pupil size | N | N | N | N | N | N | N |
| 12 | Tail position | N | N | N | N | N | N | N |
| 13 | The amount and consistency of fecal matter | N | N | N | N | N | N | N |
| 14 | Urinary frequency and urine coloration | N | N | N | N | N | N | N |
| 15 | Feed and water consumption | N | N | N | N | N | N | N |

Abbreviations: N – norm.

**Table S11.** Indicators of the rats (males and females) state during the first day after cobalt ferrite MNPs administration at a dose of 600 mg kg$^{-1}$ in the study of acute toxicity.

| № | Indicator | Watching hours | | | | | | | | | |
|---|---|---|---|---|---|---|---|---|---|---|---|
| | | 1 | 2 | 3 | 4 | 5 | 6 | 7 | 8 | 10 | 24 |
| 1 | General condition of animals | Movement disturbance, "dragging" of the hind limbs when moving, spasm of the abdominal muscles, the predominance of sleep periods, lethargy | | | | | | | | | N |
| 2 | Features of behavior | N | N | N | N | N | N | N | N | N | N |
| 3 | The intensity and nature of motor activity | Reduced motor activity | | | | | | | | | N |
| 4 | The presence and nature of seizures, coordination of movements | Periodically impaired coordination of movement | | | | | | Abs | Abs | Abs | Abs |
| 5 | Skeletal muscle tone | N | N | N | N | N | N | N | N | N | N |
| 6 | Reaction to tactile, painful, sound, and light stimuli | Weak | | | N | N | N | N | N | N | N |
| 7 | Respiratory rate | N | N | N | N | N | N | N | N | N | N |
| 8 | Heartbeat rhythm | N | N | N | N | N | N | N | N | N | N |
| 9 | Condition of the coat and skin | N | N | N | N | N | N | N | N | N | N |
| 10 | Coloring of mucous membranes | N | N | N | N | N | N | N | N | N | N |
| 11 | Pupil size | N | N | N | N | N | N | N | N | N | N |
| 12 | Tail position | Periodi-cally vertically up | | N | N | N | N | N | N | N | N |
| 13 | The amount and consistency of fecal matter | N | N | N | N | N | N | N | N | N | N |
| 14 | Urinary frequency and urine coloration | N | N | N | N | N | N | N | N | N | N |
| 15 | Feed and water consumption | Reduced | | N | N | N | N | N | N | N | N |

Abbreviations: Abs – absent; N – norm.

**Table S12.** Indicators of the rats (males and females) state during the 2-30 days after cobalt ferrite MNPs administration at a dose of 600 mg kg$^{-1}$ in the study of acute toxicity.

| № | Indicator | Watching days | | | | | | |
|---|---|---|---|---|---|---|---|---|
| | | 2 | 3 | 4 | 5 | 6… | 16… | 30 |
| 1 | General condition of animals | Male death on day 11 | | | | | | N |
| 2 | Features of behavior | N | N | N | N | N | N | N |
| 3 | The intensity and nature of motor activity | N | N | N | N | N | N | N |
| 4 | The presence and nature of seizures, coordination of movements | N | N | N | N | N | N | N |
| 5 | Skeletal muscle tone | N | N | N | N | N | N | N |
| 6 | Reaction to tactile, painful, sound, and light stimuli | N | N | N | N | N | N | N |
| 7 | Respiratory rate | N | N | N | N | N | N | N |
| 8 | Heartbeat rhythm | N | N | N | N | N | N | N |
| 9 | Condition of the coat and skin | N | N | N | N | N | N | N |
| 10 | Coloring of mucous membranes | N | N | N | N | N | N | N |
| 11 | Pupil size | N | N | N | N | N | N | N |
| 12 | Tail position | N | N | N | N | N | N | N |
| 13 | The amount and consistency of fecal matter | N | N | N | N | N | N | N |
| 14 | Urinary frequency and urine coloration | N | N | N | N | N | N | N |
| 15 | Feed and water consumption | N | N | N | N | N | N | N |

Abbreviations: N – norm.

**Table S13.** Indicators of the rats (males and females) state during the first day after cobalt ferrite MNPs administration at a dose of 106,2 mg kg$^{-1}$ in the study of acute toxicity.

| № | Indicator | Watching hours | | | | | | | | | |
|---|---|---|---|---|---|---|---|---|---|---|---|
| | | 1 | 2 | 3 | 4 | 5 | 6 | 7 | 8 | 10 | 24 |
| 1 | General condition of animals | Movement disturbance, "dragging" of the hind limbs when moving, spasm of the abdominal muscles, the predominance of sleep periods, lethargy | | | | | | | | | N |
| 2 | Features of behavior | N | N | N | N | N | N | N | N | N | N |
| 3 | The intensity and nature of motor activity | Reduced motor activity | | | | | | | | | N |
| 4 | The presence and nature of seizures, coordination of movements | Periodically impaired coordination of movement | | | | | | Abs | Abs | Abs | Abs |
| 5 | Skeletal muscle tone | N | N | N | N | N | N | N | N | N | N |
| 6 | Reaction to tactile, painful, sound, and light stimuli | Weak | | | N | N | N | N | N | N | N |
| 7 | Respiratory rate | N | N | N | N | N | N | N | N | N | N |
| 8 | Heartbeat rhythm | N | N | N | N | N | N | N | N | N | N |
| 9 | Condition of the coat and skin | N | N | N | N | N | N | N | N | N | N |
| 10 | Coloring of mucous membranes | N | N | N | N | N | N | N | N | N | N |
| 11 | Pupil size | N | N | N | N | N | N | N | N | N | N |
| 12 | Tail position | Periodi-cally vertically up | | N | N | N | N | N | N | N | N |
| 13 | The amount and consistency of fecal matter | N | N | N | N | N | N | N | N | N | N |
| 14 | Urinary frequency and urine coloration | N | N | N | N | N | N | N | N | N | N |
| 15 | Feed and water consumption | Reduced | | N | N | N | N | N | N | N | N |

Abbreviations: Abs – absent; N – norm.

**Table S14.** Indicators of the rats (males and females) state during the 2-30 days after cobalt ferrite MNPs administration at a dose of 106,2 mg kg$^{-1}$ in the study of acute toxicity.

| № | Indicator | Watching days | | | | | | |
|---|---|---|---|---|---|---|---|---|
| | | 2 | 3 | 4 | 5 | 6… | 16… | 30 |
| 1 | General condition of animals | N | N | N | N | N | N | N |
| 2 | Features of behavior | N | N | N | N | N | N | N |
| 3 | The intensity and nature of motor activity | N | N | N | N | N | N | N |

| | | | | | | | | |
|---|---|---|---|---|---|---|---|---|
| 4 | The presence and nature of seizures, coordination of movements | N | N | N | N | N | N | N |
| 5 | Skeletal muscle tone | N | N | N | N | N | N | N |
| 6 | Reaction to tactile, painful, sound, and light stimuli | N | N | N | N | N | N | N |
| 7 | Respiratory rate | N | N | N | N | N | N | N |
| 8 | Heartbeat rhythm | N | N | N | N | N | N | N |
| 9 | Condition of the coat and skin | N | N | N | N | N | N | N |
| 10 | Coloring of mucous membranes | N | N | N | N | N | N | N |
| 11 | Pupil size | N | N | N | N | N | N | N |
| 12 | Tail position | N | N | N | N | N | N | N |
| 13 | The amount and consistency of fecal matter | N | N | N | N | N | N | N |
| 14 | Urinary frequency and urine coloration | N | N | N | N | N | N | N |
| 15 | Feed and water consumption | N | N | N | N | N | N | N |

Abbreviations: N – norm.

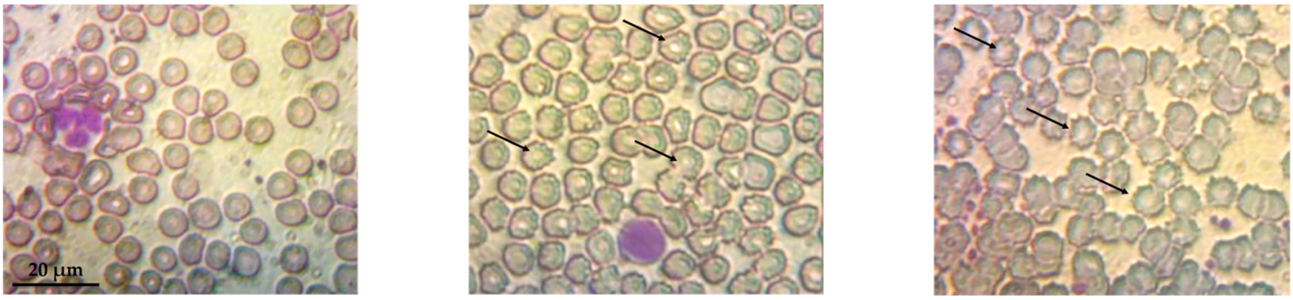


**(a)** **(b)** **(c)**

**Figure S4.** Micrographs of rats peripheral blood smears 30 days after MNPs acute toxicity experiment start. (**a**) Blood cells of female rat from intact group – type and shape of red blood cells (RBC) are without pathological changes; **(b)** Blood cells of female rat injected with 600 mg kg$^{-1}$ cobalt ferrite MNPs – pathological forms of erythrocytes (echinocytes) are observed (indicated by arrows); **(c)** Blood cells of female rat injected with 1384,6 mg kg$^{-1}$ cobalt ferrite MNPs – pathological forms of erythrocytes (echinocytes) are observed (indicated by arrows).

**Table S15.** Assessment of MNPs effect on the male mice leukocyte blood count (in the study of acute toxicity). Table cell designations: Median/Min÷Max/interquartile percentiles ($P_{25\%}$÷$P_{75\%}$).

| Dose, mg kg$^{-1}$ | Neutrophils, % | Lymphocytes, % | Monocytes, % | Eosinophils, % | Basophils, % |
|---|---|---|---|---|---|
| 0<br>(intact mice) | **16.70**<br>16.70 ÷ 16.70<br>(16.70 ÷ 16.70) | **76.20**<br>72.50 ÷ 86.60<br>(73.70 ÷ 76.60) | **2.30**<br>2.10 ÷ 6.70<br>(2.10 ÷ 2.40) | **0.00**<br>0.00 ÷ 0.00<br>(0.00 ÷ 0.00) | **0.00**<br>0.00 ÷ 0.00<br>(0.00 ÷ 0.00) |
| 0<br>(vehicle) | **8.95**<br>3.30 ÷ 14.60<br>(3.30 ÷ 14.60) | **79.10**<br>29.40 ÷ 83.90<br>(29.40 ÷ 83.90) | **4.20**<br>1.10 ÷ 67.30<br>(3.90 ÷ 6.40) | **0.05**<br>0.00 ÷ 0.40<br>(0.00 ÷ 0.25) | **0.00**<br>0.00 ÷ 0.00<br>(0.00 ÷ 0.00) |
| 230 | **16.60**<br>11.50 ÷ 19.60<br>(11.50 ÷ 19.60) | **83.10**<br>60.10 ÷ 83.60<br>(75.50 ÷ 83.40) | **3.15**<br>0.30 ÷ 5.10<br>(0.85 ÷ 5.00) | **0.00**<br>0.00 ÷ 0.10<br>(0.00 ÷ 0.05) | **0.00**<br>0.00 ÷ 0.00<br>(0.00 ÷ 0.00) |
| 1300 | **13.70**<br>9.90 ÷ 19.80<br>(9.90 ÷ 19.80) | **78.55**<br>60.40 ÷ 88.40<br>(67.95 ÷ 85.00) | **3.20**<br>1.20 ÷ 25.90<br>(1.45 ÷ 15.30) | **0.00**<br>0.00 ÷ 0.00<br>(0.00 ÷ 0.00) | **0.00**<br>0.00 ÷ 0.30<br>(0.00÷0.00) |
| 3000 | -*<br>-<br>- | -<br>-<br>- | **10.40**<br>1.90 ÷ 18.90<br>(1.90 ÷ 18.90) | **0.00**<br>0.00 ÷ 0.60<br>(0.00 ÷ 0.60) | **0.00**<br>0.00 ÷ 0.00<br>(0.00 ÷ 0.00) |
| *Kruskal-Wallis One-way* ANOVA *test* **<br>*($p_{K-W}$)* | $p_{K-W}$ > 0.05 | $p_{K-W}$ > 0.05 | $p_{K-W}$ > 0.05 | $p_{K-W}$ > 0.05 | $p_{K-W}$ > 0.05 |

Notes:
* "-" – undefined;.

** the differences are statistically insignificant (p > 0.05) compared to the "dose of 0 mg kg$^{-1}$, intact" group and "dose of 0 mg kg$^{-1}$, vehicle" group.

**Table S16.** Assessment of MNPs effect on the female mice leukocyte blood count (in the study of acute toxicity). Table cell designations: Median/Min÷Max/interquartile percentiles ($P_{25\%}$÷$P_{75\%}$).

| Dose, mg kg$^{-1}$ | Neutrophils, % | Lymphocytes, % | Monocytes, % | Eosinophils, % | Basophils, % |
|---|---|---|---|---|---|
| 0 (intact mice) | **9.20** 4.90 ÷ 13.50 (4.90 ÷ 13.50) | **83.50** 57.30 ÷ 83.90 (57.30 ÷ 83.90) | **9.10** 2.50 ÷ 37.40 (5.55 ÷ 23.50) | **0.20** 0.00 ÷ 2.90 (0.05 ÷ 1.60) | **0.10** 0.00 ÷ 0.20 (0.10 ÷ 0.10) |
| 0 (vehicle) | **10.65** 0.20 ÷ 19.40 (4.80 ÷ 15.65) | **84.30** 75.30 ÷ 88.90 (75.30 ÷ 85.30) | **5.30** 0.40 ÷ 14.10 (3.50 ÷ 10.70) | **0.10** 0.00 ÷ 1.20 (0.00 ÷ 0.70) | **0.00** 0.00 ÷ 0.30 (0.00 ÷ 0.00) |
| 230 | **14.30** 5.40 ÷ 37.40 (5.40 ÷ 37.40) | **73.60** 56.60 ÷ 79.20 (62.90 ÷ 78.60) | **11.40** 5.30 ÷ 19.20 (6.40 ÷ 17.25) | **0.20** 0.00 ÷ 0.70 (0.00 ÷ 0.70) | **0.00** 0.00 ÷ 0.60 (0.00 ÷ 0.10) |
| 1300 | **9.20** 6.30 ÷ 13.10 (6.30÷13.10) | **72.90** 56.40 ÷ 87.90 (62.65 ÷ 82.40) | **8.65** 5.60 ÷ 21.50 (6.45 ÷ 15.75) | **0.20** 0.00 ÷ 1.10 (0.00 ÷ 0.30) | **0.10** 0.00 ÷ 0.10 (0.00 ÷ 0.10) |
| 3000 | **17.15** 16.30 ÷ 18.00 (16.30 ÷ 18.00) | **65.30** 48.80 ÷ 81.80 (48.80 ÷ 81.80) | **6.70** 1.90 ÷ 32.60 (6.70 ÷ 7.40) | **0.20** 0.00 ÷ 1.00 (0.20 ÷ 0.60) | **0.00** 0.00 ÷ 0.10 (0.00 ÷ 0.00) |
| *Kruskal-Wallis One-*way ANOVA *test* ** ($p_{K-W}$) | $p_{K-W}$ > 0.05 | $p_{K-W}$ > 0.05 | $p_{K-W}$ > 0.05 | $p_{K-W}$ > 0.05 | $p_{K-W}$ > 0.05 |

Notes:.
** the differences are statistically insignificant (p > 0.05) compared to the "dose of 0 mg kg$^{-1}$, intact" group and "dose of 0 mg kg$^{-1}$, vehicle" group.

**Table S17.** Assessment of MNPs effect on the male rats leukocyte blood count (in the study of acute toxicity). Table cell designations: Median/Min÷Max/interquartile percentiles ($P_{25\%}$÷$P_{75\%}$).

| Dose, mg kg$^{-1}$ | Neutrophils, % | Lymphocytes, % | Monocytes, % | Eosinophils, % | Basophils, % |
|---|---|---|---|---|---|
| 0 (intact rats) | **23.70** 17.40÷35.20 (22.90÷27.00) | **66.50** 59.80÷74.00 (62.00÷68.90) | **3.30** 1.80÷4.70 (2.50÷4.20) | **6.30** 2.50÷6.50 (4.40÷6.40) | **0.00** 0.00÷0.00 (0.00÷0.00) |
| 0 (vehicle) | **14.60** 11.60÷24.30 (13.90÷19.10) | **70.30** 62.60÷76.90 (66.90÷76.30) | **4.50** 3.20÷6.70 (3.30÷5.40) | **7.40** 4.50÷12.40 (6.10÷9.80) | **0.00** 0.00÷0.10 (0.00÷0.10) |
| 106.2 | **25.00** 20.60÷32.80 (21.40÷30.70) | **64.90** 48.80÷69.10 (55.40÷67.80) | **5.40** 3.60÷6.40 (3.70÷5.80) | **5.90** 4.70÷14.10 (5.70÷8.10) | **0.00** 0.00÷0.10 (0.00÷0.00) |
| 600 | **26.05** 13.30÷40.80 (14.80÷38.30) | **61.80** 50.00÷79.50 (51.15÷75.40) | **6.55** 4.30÷7.20 (5.20÷7.10) | **4.00** 2.10÷5.80 (2.50÷5.45) | **0.05** 0.00÷0.10 (0.00÷0.10) |
| 1384.6 | **28.70** 10.00÷32.20 (17.10÷30.90) | **64.50** 56.70÷81.70 (58.40÷67.70) | **6.20** 1.50÷9.60 (4.40÷9.40) | **3.80** 1.40÷6.70 (3.10÷5.80) | **0.00** 0.00÷0.10 (0.00÷0.10) |
| *Kruskal-Wallis One-*way ANOVA *test* ** ($p_{K-W}$) | $p_{K-W}$ > 0.05 | $p_{K-W}$ > 0.05 | $p_{K-W}$ > 0.05 | $p_{K-W}$ > 0.05 | $p_{K-W}$ > 0.05 |

Notes:.
** the differences are statistically insignificant (p > 0.05) compared to the "dose of 0 mg kg$^{-1}$, intact" group and "dose of 0 mg kg$^{-1}$, vehicle" group.

**Table S18.** Assessment of MNPs effect on the female rats leukocyte blood count (in the study of acute toxicity). Table cell designations: Median/Min÷Max/interquartile percentiles ($P_{25\%}$÷$P_{75\%}$).

| Dose, mg kg$^{-1}$ | Neutrophils, % | Lymphocytes, % | Monocytes, % | Eosinophils, % | Basophils, % |
|---|---|---|---|---|---|
| 0<br>(intact rats) | **12.60**<br>10.90÷19.90<br>(11.65÷16.35) | **80.75**<br>70.90÷81.40<br>(75.60÷81.30) | **4.20**<br>1.60÷5.20<br>(3.00÷4.60) | **4.00**<br>3.40÷5.20<br>(3.40÷4.70) | **0.00**<br>0.00÷0.10<br>(0.00÷0.10) |
| 0<br>(vehicle) | **17.30**<br>14.50÷23.90<br>(16.00÷18.80) | **73.40**<br>64.00÷76.20<br>(72.30÷75.90) | **5.20**<br>1.80÷9.70<br>(4.40÷5.50) | **3.40**<br>2.60÷7.70<br>(3.20÷4.10) | **0.00**<br>0.00÷0.10<br>(0.00÷0.00) |
| 106.2 | **16.40**<br>11.20÷26.30<br>(12.20÷24.50) | **67.60**<br>62.00÷80.60<br>(66.20÷80.40) | **4.80**<br>3.00÷6.80<br>(3.60÷5.30) | **4.50**<br>2.80÷9.20<br>(3.80÷8.70) | **0.00**<br>0.00÷0.10<br>(0.00÷0.00) |
| 600 | **16.00**<br>6.40÷28.80<br>(10.50÷17.60) | **77.40**<br>63.90÷88.70<br>(73.60÷80.30) | **3.50**<br>2.70÷4.60<br>(3.30÷4.30) | **3.30**<br>2.10÷5.70<br>(3.00÷4.10) | **0.00**<br>0.00÷0.10<br>(0.00÷0.10) |
| 1384.6 | **16.25**<br>9.80÷46.70<br>(12.70÷31.80) | **73.40**<br>41.40÷82.90<br>(56.95÷78.60) | **6.80**<br>5.00÷8.60<br>(5.80÷7.80) | **3.20**<br>2.20÷4.00<br>(2.65÷3.65) | **0.00**<br>0.00÷0.10<br>(0.00÷0.05) |
| *Kruskal-Wallis One-*way ANOVA *test* ** *($p_{K-W}$)* | $p_{K-W}$ *> 0.05* | $p_{K-W}$ *> 0.05* | $p_{K-W}$ *< 0.05* | $p_{K-W}$ *> 0.05* | $p_{K-W}$ *> 0.05* |

Notes:.
** the differences are statistically insignificant (p > 0.05) or significant (p < 0.05) compared to the "dose of 0 mg kg$^{-1}$, intact" group and "dose of 0 mg kg$^{-1}$, vehicle" group.

**Table S19.** Assessment of MNPs effect on the male mice blood biochemical parameters (in the study of acute toxicity). Table cell designations: Median/Min÷Max/interquartile percentiles ($P_{25\%}$÷$P_{75\%}$).

| Dose, mg kg$^{-1}$ | Venous blood plasma glu-cose, mM L$^{-1}$ | Total biliru-bin, mM L$^{-1}$ | Total cho-leste-rol, mM L$^{-1}$ | Total protein, g L$^{-1}$ | Creatini-ne (Enzyma-tic-IDSM), μM L$^{-1}$ | Alanine amino-transferase (ALT), U L$^{-1}$ | Aspartate amino-transfer-ase (AST), U L$^{-1}$ | Gamma-glu-tamyl transfe-rase (GGT), U L$^{-1}$ |
|---|---|---|---|---|---|---|---|---|
| 0<br>(intact mice) | **8.56**<br>5.36÷11.55<br>7.69÷9.54 | **5.50**<br>4.40÷6.00<br>4.70÷6.00 | **2.23**<br>2.05÷2.86<br>2.21÷2.68 | **68.90**<br>67.50÷76.40<br>68.90÷69.80 | **8.00**<br>6.00÷21.00<br>7.00÷10.00 | **51.25**<br>48.79÷61.99<br>49.96÷56.68 | **122.34**<br>105.93÷139.44<br>110.56÷134.46 | **0.00**<br>0.00÷1.65<br>0.00÷0.86 |
| 0<br>(vehic-le) | **9.71**<br>7.98÷11.10<br>8.52÷10.50 | **5.10**<br>4.50÷5.80<br>4.80÷5.50 | **2.18**<br>2.08÷2.92<br>2.16÷2.27 | **73.20**<br>65.70÷75.10<br>68.60÷73.20 | **5.00**<br>3.00÷10.00<br>4.00÷9.00 | **57.71**<br>51.13÷101.00<br>55.78÷58.49 | **129.29**<br>100.77÷196.54<br>113.04÷164.91 | **-***<br>-<br>- |
| 230 | **8.45**<br>6.47÷12.52<br>6.82÷10.52 | **5.05**<br>3.80÷6.30<br>3.80÷6.30 | **2.90**<br>2.65÷3.14<br>2.65÷3.14 | **86.50**<br>85.70÷87.30<br>85.70÷87.30 | **7.50**<br>7.00÷8.00<br>7.00÷8.00 | **-***<br>-<br>- | **185. 54**<br>185.54÷185.54<br>185.54÷185.54 | **0.00**<br>0.00÷0.00<br>0.00÷0.00 |
| *Kruskal-Wallis test ** ($p_{K-W}$)* | > 0.05 | > 0.05 | > 0.05 | < 0.05 | > 0.05 | > 0.05 | > 0.05 | > 0.05 |
| 1300 | **6.12**<br>5.54÷6.47<br>5.84÷6.41 | **5.05**<br>4.30÷7.20<br>4.60÷6.20 | **2.55**<br>2.26÷4.03<br>2.34÷3.35 | **85.75**<br>73.60÷92.90<br>79.35÷89.65 | **7.50**<br>5.00÷9.00<br>6.00÷8.50 | **49.75**<br>49.75÷49.75<br>49.75÷49.75 | **120.98**<br>120.98÷120.98<br>120.98÷120.98 | **0.66**<br>0.00÷14.11<br>0.21÷7.51 |
| *Kruskal-Wallis test ** ($p_{K-W}$)* | > 0.05 | > 0.05 | > 0.05 | < 0.05 | > 0.05 | > 0.05 | > 0.05 | > 0.05 |
| 3000 | **6.05**<br>5.52÷8.32<br>5.71÷7.27 | **5.00**<br>3.80÷5.60<br>3.80÷5.60 | **1.56**<br>1.28÷2.33<br>1.28÷2.33 | **69.60**<br>68.90÷72.40<br>68.90÷72.40 | **8.00**<br>8.00÷8.00<br>8.00÷8.00 | **44.55**<br>34.60÷51.68<br>34.60÷51.68 | **126.40**<br>125.42÷127.37<br>125.42÷127.37 | **0.00**<br>0.00÷0.00<br>0.00÷0.00 |

| | | | | | | | | |
|---|---|---|---|---|---|---|---|---|
| *Kruskal-Wallis test *** ($p_{K-W}$)* | > 0.05 | > 0.05 | > 0.05 | > 0.05 | > 0.05 | > 0.05 | > 0.05 | > 0.05 |

Notes:.
* "-" – undefined;.
** the differences are statistically significant (p < 0.05) compared to the "dose of 0 mg kg$^{-1}$, intact" group and "dose of 0 mg kg$^{-1}$, vehicle" group.
*** the differences are statistically insignificant (p > 0.05) compared to the "dose of 0 mg kg$^{-1}$, intact" group and "dose of 0 mg kg$^{-1}$, vehicle" group.

**Table S20.** Assessment of MNPs effect on the female mice blood biochemical parameters (in the study of acute toxicity). Table cell designations: Median/Min÷Max/interquartile percentiles ($P_{25\%}$÷$P_{75\%}$).

| Dose, mg kg$^{-1}$ | Venous blood plasma glucose, mM L$^{-1}$ | Total bilirubin, mM L$^{-1}$ | Total cholesterol, mM L$^{-1}$ | Total protein, g L$^{-1}$ | Creatinine (Enzymatic IDSM), μM L$^{-1}$ | Alanine aminotransferase (ALT), U L$^{-1}$ *** | Aspartate aminotransferase (AST), U L$^{-1}$ *** | Gamma-glutamyl transferase (GGT), U L$^{-1}$ |
|---|---|---|---|---|---|---|---|---|
| 0 (intact mice) | **9.21** 7.14÷12.80 7.66÷11.52 | **4.60** 4.40÷6.10 4.40÷6.10 | **2.48** 1.71÷2.82 1.71÷2.82 | **87.20** 80.40÷91.30 80.40÷91.30 | **8.00** 1.00÷9.00 1.00÷9.00 | **-*** | **-*** | **0.00** 0.00÷4.58 0.00÷4.58 |
| 0 (vehicle) | **9.52** 5.78÷11.52 6.84÷10.38 | **4.90** 3.90÷5.20 4.50÷5.10 | **1.94** 1.49÷2.61 1.85÷2.02 | **60.30** 59.00÷81.40 60.00÷69.00 | **2.00** 0.00÷5.00 1.00÷5.00 | **52.72** 45.94÷59.20 49.55÷55.38 | **110.36** 105.01÷148.89 108.24÷142.15 | **0.00** 0.00÷0.00 0.00÷0.00 |
| 230 | **6.84** 5.54÷11.82 5.54÷11.82 | **6.20** 6.10÷6.40 6.10÷6.40 | **1.49** 1.47÷2.41 1.47÷2.41 | **87.50** 74.20÷98.60 74.20÷98.60 | **6.00** 6.00÷8.00 6.00÷8.00 | **76.71** 52.42÷101.00 52.42÷101.00 | **186.25** 101.00÷271.49 101.00÷271.49 | **1.52** 0.00÷3.03 0.00÷3.03 |
| *Kruskal-Wallis test *** ($p_{K-W}$)* | > 0.05 | > 0.05 | > 0.05 | > 0.05 | > 0.05 | > 0.05 | > 0.05 | > 0.05 |
| 1300 | **6.01** 5.70÷7.90 5.75÷7.06 | **6.15** 5.70÷6.90 5.90÷6.55 | **1.92** 1.52÷2.34 1.68÷2.17 | **88.65** 85.10÷105.1 85.30÷98.45 | **7.50** 0.00÷10.00 3.50÷9.00 | **54.38** 54.38÷54.38 54.38÷54.38 | **151.04** 151.04÷151.04 151.04÷151.04 | **0.00** 0.00÷0.00 0.00÷0.00 |
| *Kruskal-Wallis test ** ($p_{K-W}$)* | < 0.05 | > 0.05 | > 0.05 | > 0.05 | > 0.05 | > 0.05 | > 0.05 | > 0.05 |
| 3000 | **5.56** 4.98÷6.20 5.20÷5.95 | **4.65** 3.90÷6.00 4.00÷5.60 | **1.78** 1.32÷2.16 1.38÷2.15 | **64.70** 59.30÷75.60 60.70÷71.45 | **9.00** 7.00÷21.00 8.00÷15.00 | **59.47** 48.19÷101.00 48.19÷101.00 | **143.84** 114.12÷151.34 114.12÷151.34 | **0.00** 0.00÷0.73 0.00÷0.37 |
| *Kruskal-Wallis test ** ($p_{K-W}$)* | < 0.05 | > 0.05 | > 0.05 | < 0.05 | > 0.05 | > 0.05 | > 0.05 | > 0.05 |

Notes:.
* "-" – undefined;.
** the differences are statistically significant (p < 0.05) compared to the "dose of 0 mg kg$^{-1}$, intact" group and "dose of 0 mg kg$^{-1}$, vehicle" group.
*** the differences are statistically insignificant (p > 0.05) compared to the "dose of 0 mg kg$^{-1}$, intact" group and "dose of 0 mg kg$^{-1}$, vehicle" group.

**Table S21.** Assessment of MNPs effect on the male rats blood biochemical parameters (in the study of acute toxicity). Table cell designations: Median/Min÷Max/interquartile percentiles ($P_{25\%}$÷$P_{75\%}$).

| Dose, mg kg$^{-1}$ | Venous blood plasma glu-cose, mM L$^{-1}$ | Total biliru-bin, mM L$^{-1}$ | Total cho-leste-rol, mM L$^{-1}$ | Total protein, g L$^{-1}$ | Creatini-ne (Enzyma-tic-IDSM), μM L$^{-1}$ | Alanine amino-transferase (ALT), U L$^{-1}$ | Aspartate amino-transfer-ase (AST), U L$^{-1}$ | Gamma-glu-tamyl transfe-rase (GGT), U L$^{-1}$ |
|---|---|---|---|---|---|---|---|---|
| 0<br>(intact<br>mice) | **7.55**<br>6.52÷8.35<br>7.42÷7.95 | **7.80**<br>6.60÷8.80<br>6.80÷8.10 | **1.81**<br>1.67÷2.06<br>1.74÷2.01 | **82.60**<br>77.80÷89.00<br>79.80÷83.80 | **24.00**<br>21.0÷37.0<br>21.0÷29.0 | **88.75**<br>61.23÷102.34<br>77.84÷94.56 | **144.10**<br>126.77÷162.34<br>138.57÷150.57 | **1.88**<br>0.63÷3.74<br>1.88÷3.15 |
| 0<br>(vehic-le) | **8.13**<br>6.25÷9.78<br>7.82÷8.96 | **7.60**<br>5.70÷9.70<br>5.90÷8.00 | **1.99**<br>1.75÷2.11<br>1.78÷2.00 | **81.70**<br>80.60÷83.50<br>81.30÷83.20 | **27.00**<br>12.0÷37.0<br>21.0÷27.0 | **64.18**<br>58.85÷90.10<br>61.44÷64.94 | **136.08**<br>114.01÷147.86<br>123.51÷147.48 | **3.38**<br>0.27÷4.50<br>2.98÷4.33 |
| 106.2 | **7.29**<br>6.21÷8.93<br>7.02÷8.25 | **6.60**<br>2.90÷7.50<br>5.30÷7.00 | **1.89**<br>1.62÷2.53<br>1.85÷2.15 | **85.00**<br>75.00÷86.60<br>80.50÷85.60 | **28.00**<br>25.0÷32.0<br>27.0÷30.0 | **77.30**<br>66.83÷96.14<br>70.98÷93.99 | **132.90**<br>107.09÷135.51<br>125.42÷134.82 | **2.93**<br>2.27÷4.57<br>2.45÷3.10 |
| *Kruskal-Wallis test *** ($p_{K-W}$)* | > 0.05 | > 0.05 | > 0.05 | > 0.05 | > 0.05 | > 0.05 | > 0.05 | > 0.05 |
| 600 | **9.38**<br>8.21÷11.32<br>8.73÷10.41 | **1.45**<br>0.50÷2.90<br>0.85÷2.30 | **1.24**<br>1.13÷1.32<br>1.17÷1.29 | **84.35**<br>79.90÷87.00<br>81.45÷86.3 | **21.50**<br>17.0÷27.0<br>18.5÷25.0 | **53.86**<br>50.66÷64.27<br>50.68÷60.64 | **137.22**<br>114.78÷146.41<br>123.77÷144.05 | **6.19**<br>0.82÷13.00<br>2.88÷10.22 |
| *Kruskal-Wallis test ** ($p_{K-W}$)* | < 0.05 | < 0.05 | < 0.05 | > 0.05 | > 0.05 | < 0.05 | > 0.05 | > 0.05 |
| 1384.6 | **10.43**<br>9.81÷11.87<br>10.28÷11.4 | **1.30**<br>0.00÷1.60<br>1.10÷1.50 | **1.47**<br>1.16÷1.80<br>1.22÷1.56 | **86.00**<br>83.20÷92.20<br>84.70÷87.00 | **23.00**<br>18.0÷32.0<br>19.0÷23.0 | **58.44**<br>28.73÷67.27<br>54.65÷66.96 | **95.09**<br>68.35÷137.01<br>82.60÷105.97 | **0.00**<br>0.00÷7.37<br>0.00÷5.53 |
| *Kruskal-Wallis test ** ($p_{K-W}$)* | < 0.05 | < 0.05 | < 0.05 | > 0.05 | > 0.05 | < 0.05 | < 0.05 | > 0.05 |

Notes:.
** the differences are statistically significant ($p < 0.05$) compared to the "dose of 0 mg kg$^{-1}$, intact" group and "dose of 0 mg kg$^{-1}$, vehicle" group.
*** the differences are statistically insignificant ($p > 0.05$) compared to the "dose of 0 mg kg$^{-1}$, intact" group and "dose of 0 mg kg$^{-1}$, vehicle" group.

**Table S22.** Assessment of MNPs effect on the female rats blood biochemical parameters (in the study of acute toxicity). Table cell designations: Median/Min÷Max/interquartile percentiles ($P_{25\%}$÷$P_{75\%}$).

| Dose, mg kg$^{-1}$ | Venous blood plasma glu-cose, mM L$^{-1}$ | Total biliru-bin, mM L$^{-1}$ | Total cho-leste-rol, mM L$^{-1}$ | Total protein, g L$^{-1}$ | Creatini-ne (Enzyma-tic-IDSM), μM L$^{-1}$ | Alanine amino-transferase (ALT), U L$^{-1}$ | Aspartate amino-transfer-ase (AST), U L$^{-1}$ | Gamma-glu-tamyl transfe-rase (GGT), U L$^{-1}$ |
|---|---|---|---|---|---|---|---|---|
| 0<br>(intact<br>mice) | **7.59**<br>6.71÷8.41<br>6.85÷8.12 | **7.20**<br>6.30÷7.40<br>7.10÷7.30 | **1.76**<br>1.65÷1.92<br>1.75÷1.86 | **86.10**<br>83.90÷88.70<br>86.10÷88.20 | **22.00**<br>7.00÷24.00<br>19.0÷23.0 | **75.19**<br>65.11÷85.78<br>71.51÷82.63 | **149.78**<br>135.48÷151.25<br>141.92÷150.90 | **2.62**<br>1.62÷2.85<br>2.60÷2.69 |
| 0<br>(vehic-le) | **8.73**<br>6.77÷8.95<br>7.90÷8.74 | **6.60**<br>4.60÷6.70<br>5.60÷6.60 | **1.78**<br>1.73÷2.17<br>1.76÷2.10 | **82.30**<br>80.70÷88.50<br>81.10÷85.70 | **20.00**<br>14.0÷22.0<br>17.0÷21.0 | **67.16**<br>63.89÷82.09<br>64.80÷67.78 | **143.63**<br>116.40÷155.30<br>126.83÷154.51 | **0.33**<br>0.00÷1.83<br>0.25÷1.53 |
| 106.2 | **7.95**<br>6.11÷8.23<br>7.25÷7.98 | **6.60**<br>6.10÷8.10<br>6.50÷6.80 | **1.59**<br>1.32÷1.90<br>1.51÷1.82 | **80.60**<br>79.40÷87.40<br>79.90÷81.90 | **21.00**<br>19.0÷25.0<br>21.0÷23.0 | **58.87**<br>49.50÷83.50<br>54.40÷60.76 | **132.21**<br>126.16÷146.80<br>129.17÷137.29 | **1.15**<br>0.00÷2.71<br>0.75÷2.38 |

| | | | | | | | | |
|---|---|---|---|---|---|---|---|---|
| *Kruskal-Wallis test ** ($p_{K\text{-}W}$)* | > 0.05 | > 0.05 | < 0.05 | > 0.05 | > 0.05 | > 0.05 | < 0.05 | > 0.05 |
| 600 | **8.91**<br>7.03÷10.44<br>7.87÷9.62 | **0.80**<br>0.00÷2.30<br>0.00÷1.50 | **1.37**<br>0.78÷1.95<br>1.06÷1.55 | **90.50**<br>82.70÷95.20<br>85.70÷90.60 | **26.00**<br>14.0÷32.0<br>17.0÷27.0 | **50.71**<br>35.73÷77.40<br>49.08÷55.03 | **146.73**<br>126.51÷161.61<br>139.31÷150.48 | **1.85**<br>0.00÷5.58<br>0.00÷2.23 |
| *Kruskal-Wallis test ** ($p_{K\text{-}W}$)* | > 0.05 | < 0.05 | > 0.05 | > 0.05 | > 0.05 | < 0.05 | > 0.05 | > 0.05 |
| 1384.6 | **11.60**<br>10.6÷12.15<br>11.09÷11.9 | **2.45**<br>1.40÷3.40<br>1.60÷3.25 | **1.65**<br>1.60÷2.32<br>1.62÷2.00 | **91.10**<br>86.80÷103.9<br>87.60÷98.85 | **26.00**<br>14.0÷34.0<br>18.5÷31.5 | **62.13**<br>42.37÷83.73<br>49.45÷75.73 | **94.05**<br>72.97÷121.52<br>80.91÷110.39 | **4.07**<br>0.00÷12.48<br>0.78÷9.53 |
| *Kruskal-Wallis test ** ($p_{K\text{-}W}$)* | < 0.05 | < 0.05 | > 0.05 | > 0.05 | > 0.05 | > 0.05 | < 0.05 | > 0.05 |

Notes:.
** the differences are statistically significant ($p < 0.05$) compared to the "dose of 0 mg $kg^{-1}$. intact" group and "dose of 0 mg $kg^{-1}$, vehicle" group.

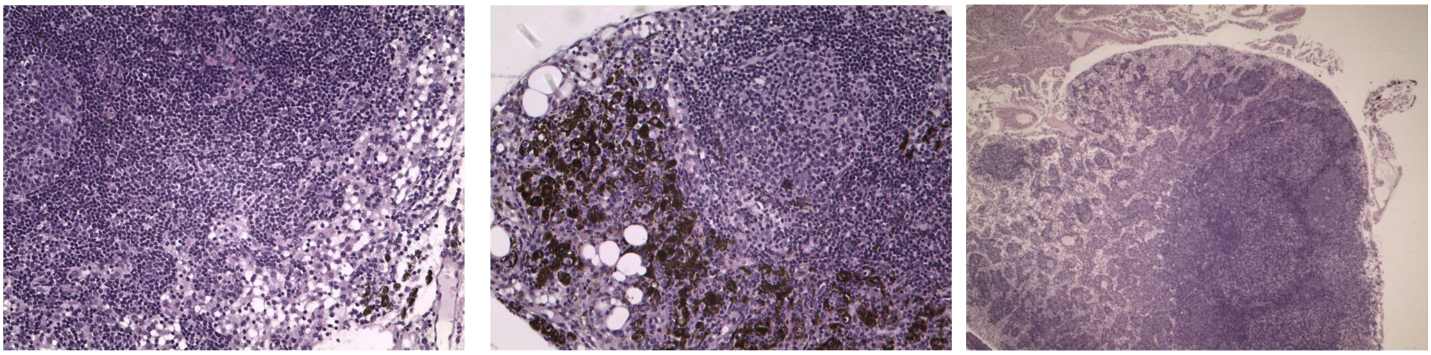

**(a)** **(b)** **(c)**

**Figure S5.** Micrographs of mice lymph node after MNPs injection. **(a)** Administration of MNPs at minimal dose. Cortex of lymph node. A site of a lymphoid nodule with a reactive center. Cells impregnated with MNPs (dark) in the sinus system; **(b)** Injection of MNPs at intermediat dose. Internodal distribution of impregnated cells. The impregnated cells are large, polygonal in shape, their nuclei are free of MNPs; **(c)** Administration of MNPs at maximum dose. Cortex and medulla. Slight impregnation of extraorganic connective tissue with MNPs. Hematoxylin-eosin staining. No visible changes in lymph node structure are found after MNPs injection at all tested doses: cortex and medulla of this organ are distinguishable; the reactive centers of lymphoid nodules as well as immunoblasts in the lymphoid tissue of the brain cords are clearly defined.

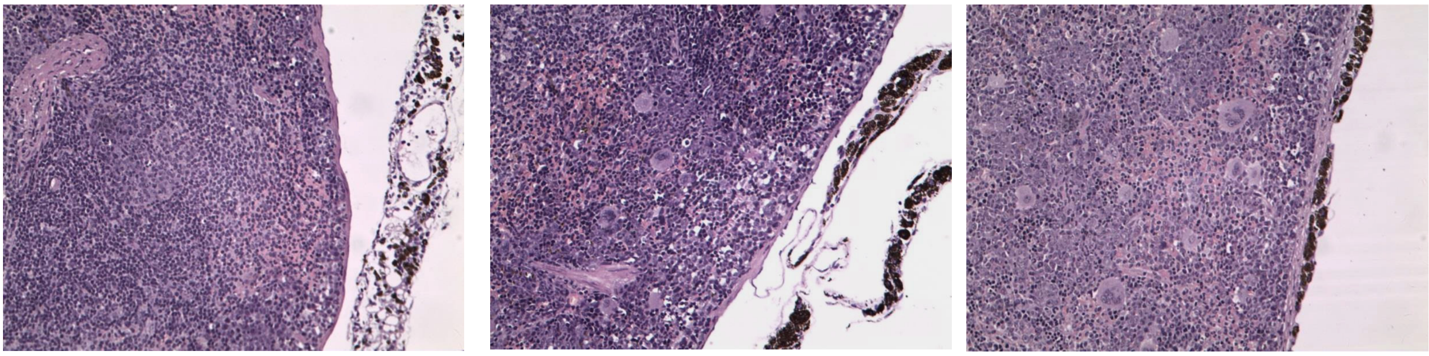

**(a)** **(b)** **(c)**

**Figure S6.** Micrographs of mice spleen after MNPs injection. **(a)** Administration of MNPs at minimal dose. White and red spleen pulp. The connective tissue structure located outside the organ is impregnated with MNPs (dark); **(b)** Injection of MNPs at intermediat dose. Impregnation of the connective tissue capsule cells with MNPs. Megakaryocytes in the red pulp; **(c)** Administration of

MNPs at maximum dose. Impregnation of the connective tissue capsule cells with MNPs. The red pulp contains a large number of megakaryocytic cells. Hematoxylin-eosin staining. The organ structure is not changed: the structures of the white pulp, such as periarterial lymphoid sheaths and lymphoid nodules, are distinguishable. The red pulp is abundantly supplied with blood. Signs of antigen-independent hematopoiesis (megakaryocytes in the red pulp) and immunopoiesis are distinguishable. The reactive centers of the lymphoid nodules are clearly defined.

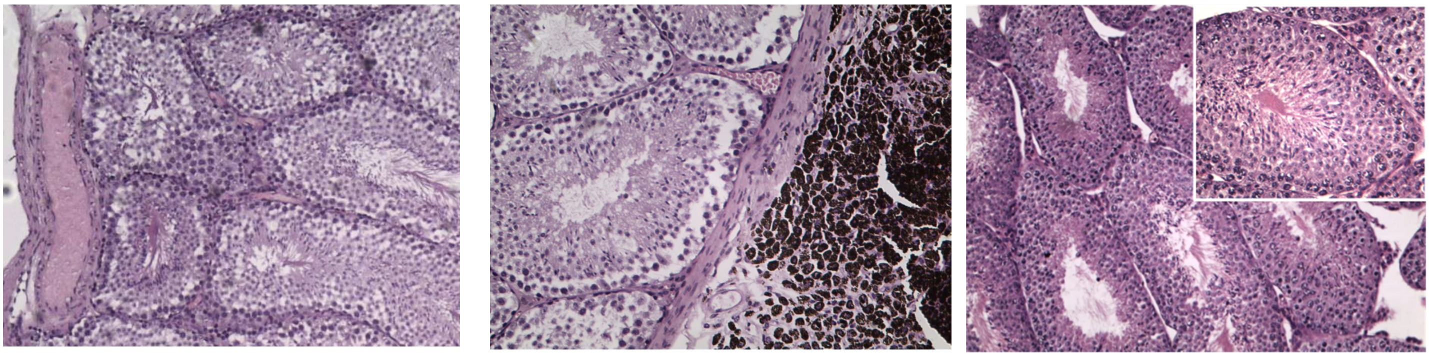

**(a)** **(b)** **(c)**

**Figure S7.** Micrographs of mice testis after MNPs injection. **(a)** Administration of MNPs at minimal dose; **(b)** Injection of MNPs at intermediat dose; **(c)** Administration of MNPs at maximum dose. Insertion demonstrates the convoluted tubules of the testis, in which spermatogonia, spermatocytes, and spermatids are clearly differentiated. Hematoxylin-eosin staining.

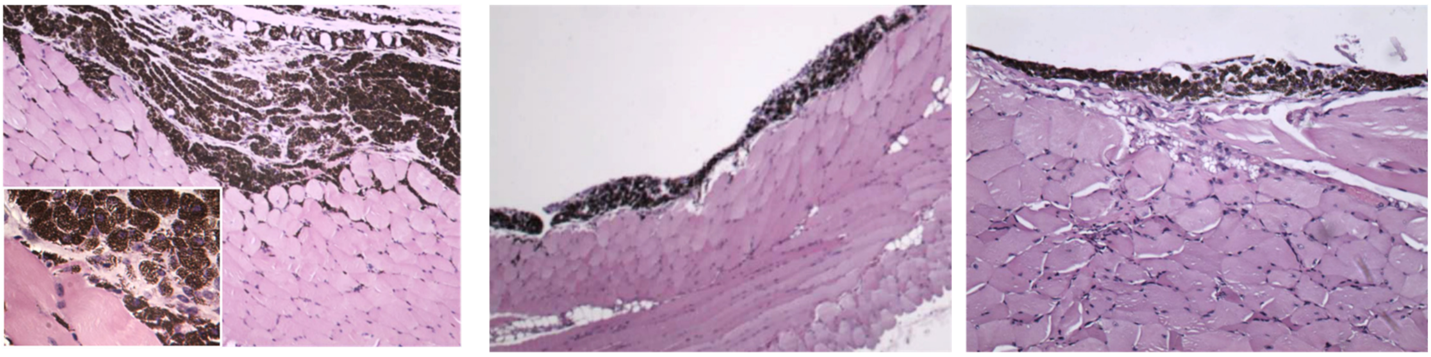

**(a)** **(b)** **(c)**

**Figure S8.** Micrographs of mice injection site after MNPs administration. **(a)** Administration of MNPs at minimal dose; **(b)** Injection of MNPs at intermediat dose; **(c)** Administration of MNPs at maximum dose. Connective tissue structures are clearly impregnated with dark particles. Insertion in **(a)** demonstrates impregnated connective tissue cells. Hematoxylin-eosin staining.

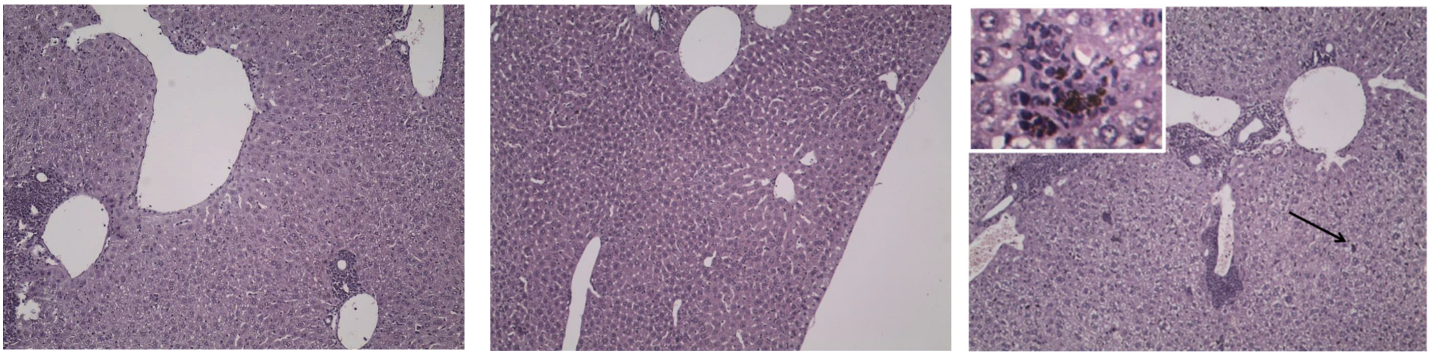

**(a)** **(b)** **(c)**

**Figure S9.** Micrographs of mice liver after MNPs injection. **(a)** Administration of MNPs at minimal dose. Lobular organization of the parenchyma. Radial arrangement of hepatocytes beams; **(b)** Injection of MNPs at intermediat dose. Hepatocytes are without pronounced changes, have 1-2 nuclei, form hepatic tracts; **(c)** Administration of MNPs at maximum dose. There are small round-cell

infiltrates inside the lobules, single cells are impregnated with MNPs (arrow and insertion). Hematoxylin-eosin staining. The organ-specific structure of the liver is not changed due to MNPs administration at all investigated doses: lobularity of the parenchyma is expressed. The hepatocyte beams are oriented radially from the central vein. Hepatocytes containe 1-2 nuclei and oxyphilically stained cytoplasm. Portal zones containe small round-cell infiltrates, while single cells are impregnated with MNPs.

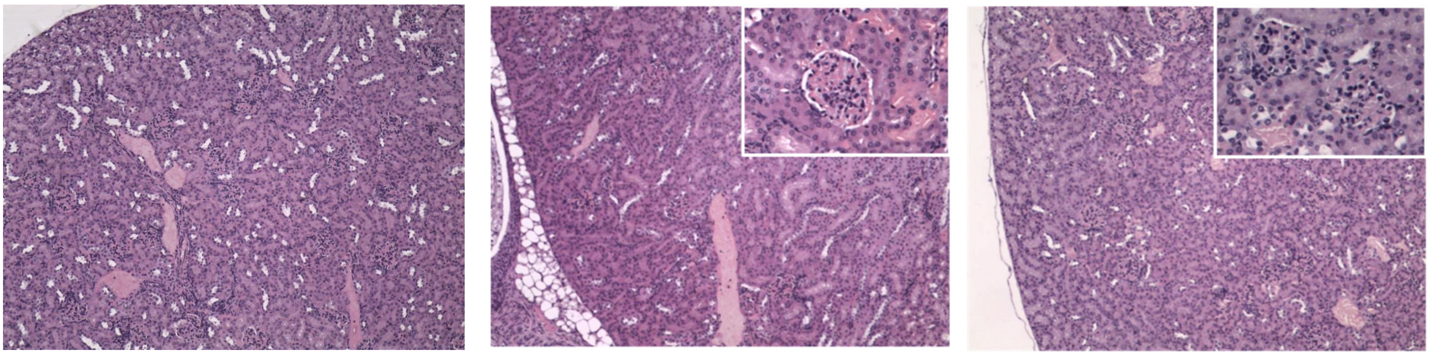

**(a)** **(b)** **(c)**

**Figure S10:** Micrographs of mice kidneys after MNPs injection. **(a)** Administration of MNPs at minimal dose; **(b)** Injection of MNPs at intermediat dose; **(c)** Administration of MNPs at maximum dose. Hematoxylin-eosin staining. No organ changes are found after animals' injection with MNPs at all tested concentrations: the structure is clearly subdivided into cortex and medulla. Arc vessels are full-blooded. The renal corpuscles include the vascular glomerulus. The cavity of the renal corpuscle is not expanded. The convoluted tubules of the cortex are lined with prismatic epithelium without visible changes. The renal tubules of the medulla are without visible changes.

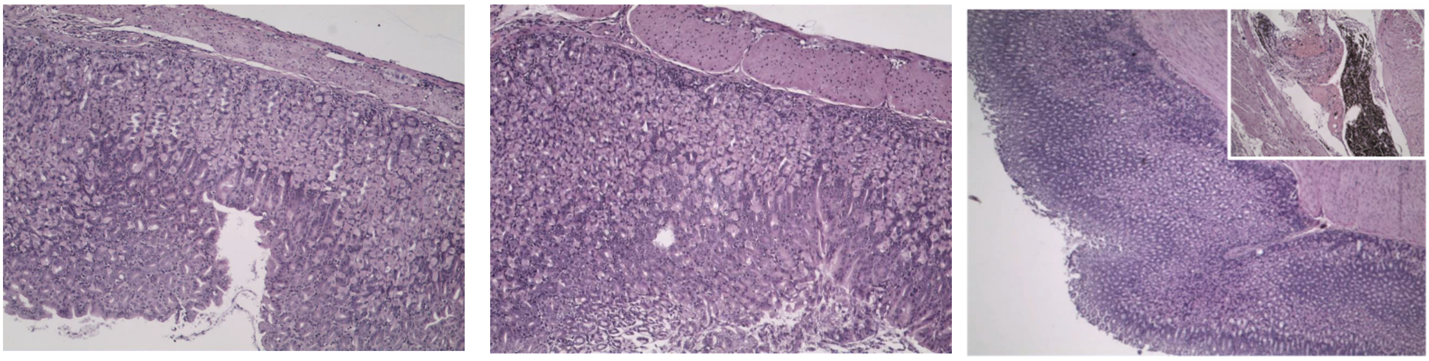

**(a)** **(b)** **(c)**

**Figure S11:** Micrographs of mice stomach after MNPs injection. **(a)** Administration of MNPs at minimal dose; **(b)** Injection of MNPs at intermediat dose; **(c)** Administration of MNPs at maximum dose. Layered organization of the mucosa, submucosa, muscular, and serous membranes. Insertion in **(c)** demonstrates the MNPs impregnation of mesentery connective tissue structures. Hematoxylin-eosin staining. None of the MNPs doses lead to significant changes in organ morphology: the layered organization of the mucosa, submucosa, muscle, and serous membranes is preserved. The gastric pits are not deep, lined with prismatic epithelium. The proper glands of the mucous membrane containe numerous parietal cells, in the bottom parts – small, basophilic stained cells. The lamina propria of the mucous membrane in the region of the bottom parts of the glands containe numerous full-blooded vessels. Serous membrane is unchanged.

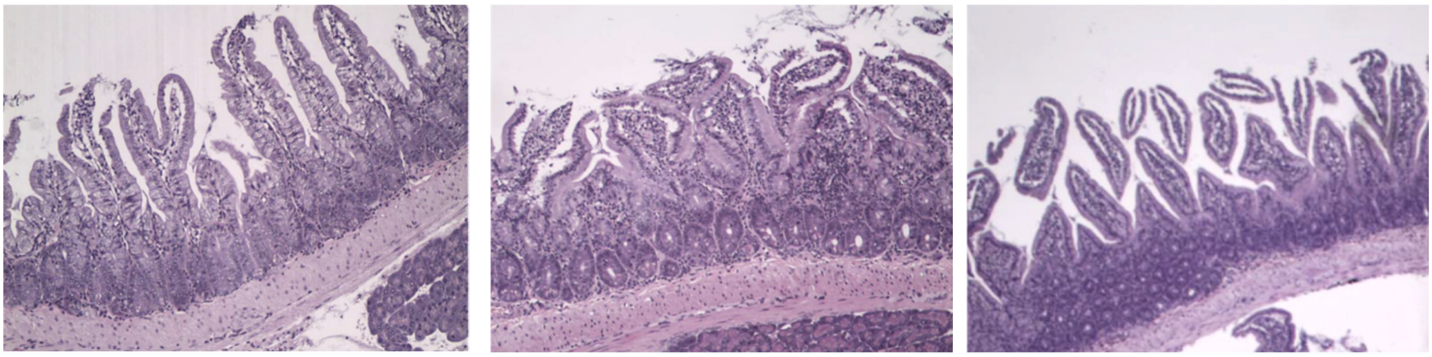

**(a)** **(b)** **(c)**

**Figure S12:** Micrographs of mice intestines after MNPs injection. **(a)** Administration of MNPs at minimal dose; **(b)** Injection of MNPs at intermediat dose; **(c)** Administration of MNPs at maximum dose. Hematoxylin-eosin staining. The structure of the intestines is not changed due to MNPs administration: the intestinal wall is formed by mucosa, submucosa, muscular, and serous membranes, which have a typical layer-by-layer organization. Folds and crypts are found in the relief. The epithelial lamina of the mucosa containe numerous goblet cells.

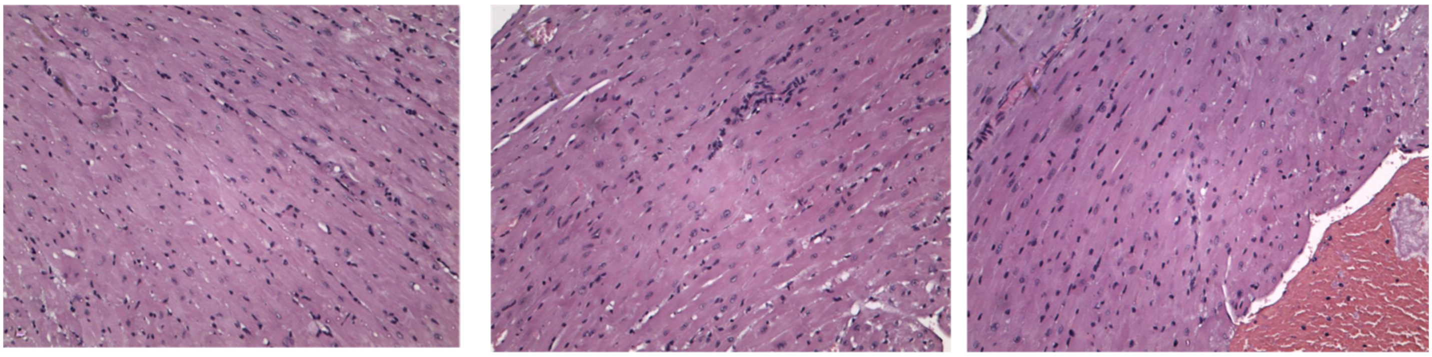

**(a)** **(b)** **(c)**

**Figure S13:** Micrographs of mice heart after MNPs injection. **(a)** Administration of MNPs at minimal dose; **(b)** Injection of MNPs at intermediat dose; **(c)** Administration of MNPs at maximum dose. Myocardium: working cardiomyocytes. Hematoxylin-eosin staining. No visible changes in the structure of the heart are found after MNPs injection: the myocardium is represented by functional fibers formed by working cardiomyocytes. Between the cardiomyocytes, there are thin layers of connective tissue, which are supplied with blood by small blood capillaries of the somatic type.

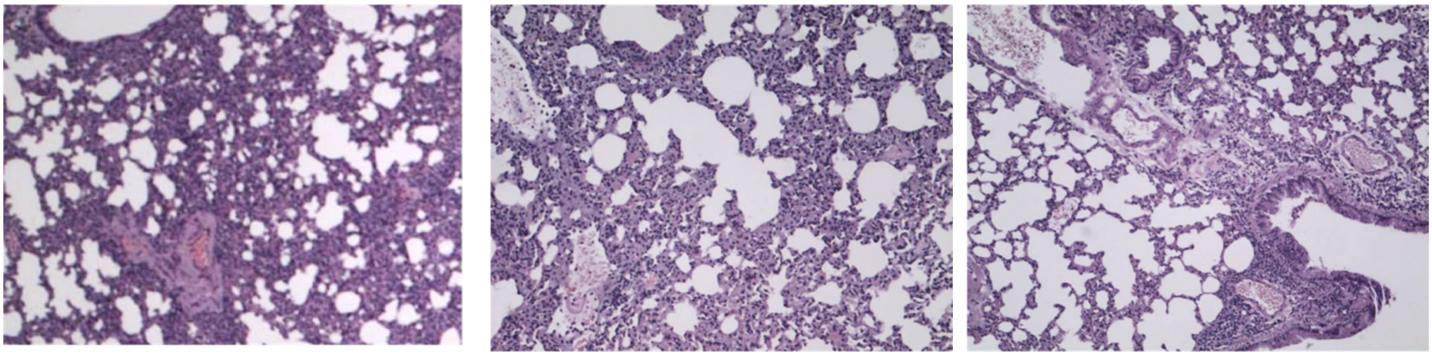

**(a)** **(b)** **(c)**

**Figure S14:** Micrographs of mice lung after MNPs injection. **(a)** Administration of MNPs at minimal dose; **(b)** Injection of MNPs at intermediat dose; **(c)** Administration of MNPs at maximum dose. Broncho-associated lymphoid tissue is visualized in the wall of the bronchi. Hematoxylin-eosin staining. No visible changes are found in the respiratory section due to MNPs administration at all investigated doses: bronchi of various sizes are determined in the sections of the lungs. In the middle bronchi, mucosa, submucosa, fibrocartilaginous and adventitious membranes are differentiated. In the mucous and submucosal membranes, lymphoid tissue is visualized, located diffusely or in the form of lymphoid nodules.

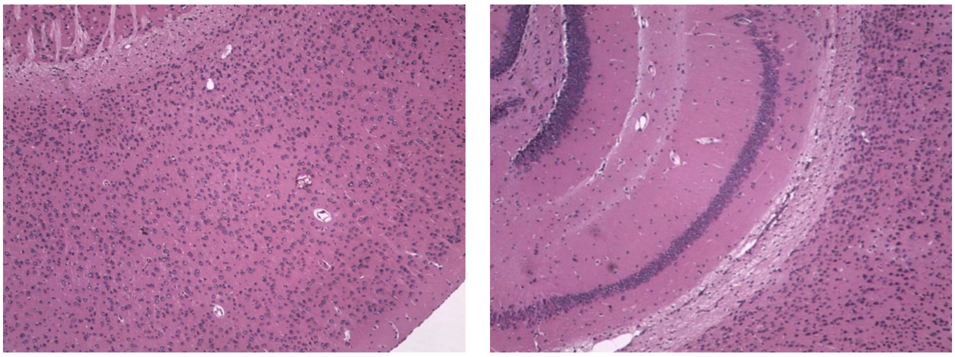

**(a)** **(b)**

**Figure S15:** Micrographs of mice brain after MNPs injection at intermediat dose. The identical results are obtained for other doses. **(a)** The neocortex is of the sensorimotor type. Molecular, outer and inner granular layers are clearly expressed. The pyramidal layers are poorly expressed. White matter. **(b)** Hippocampus. Neuroarchitectonics has a characteristic organization. Hematoxylin-eosin staining. The studied parts of the brain are not changed: the neocortex has a distinct layered organization. Neural populations characteristic of 1-6 cortex layers have typical localization. Diffuse or focal changes are not identified. The hippocampus has a characteristic structure, neuro- and glioarchitectonics are without features. The pia mater is unchanged.

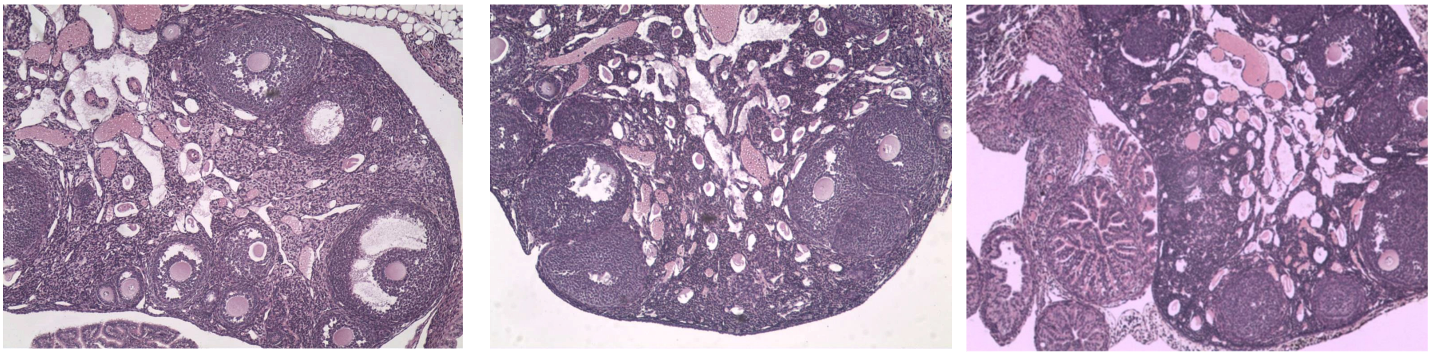

**(a)** **(b)** **(c)**

**Figure S16:** Micrographs of mice ovaries after MNPs injection. **(a)** Administration of MNPs at minimal dose; **(b)** Injection of MNPs at intermediat dose; **(c)** Administration of MNPs at maximum dose. Hematoxylin-eosin staining. In the cortex, primordial, primary, secondary, mature follicles, atretic formations, and yellow bodies are found. Secondary follicles containe developed follicular epithelium. The ovocyte in them is surrounded by a zona pellucida. Two layers of theca are differentiated. Primordial follicles are few in number. Atretic follicles are found in large numbers in the deep layers of the cortex. The vessels of the medulla are dilated, filled with blood. Oviduct wall is without visible changes.

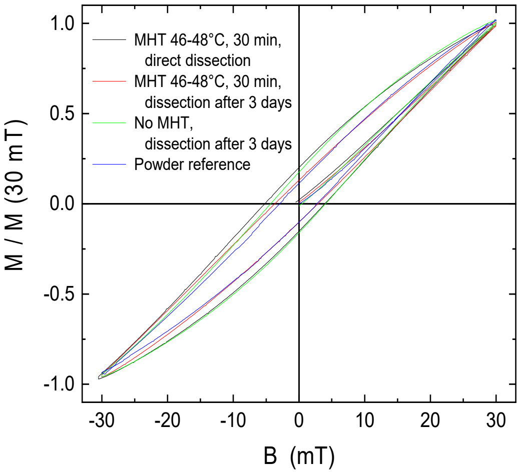


**Figure S17.** Minor magnetic hysteresis in B = ± 30 mT at T = 45 °C. All loops are normalized to the magnetization at 30 mT.
